# Engineering *Lactococcus lactis* as a multi-stress tolerant biosynthetic chassis by deleting the prophage-related fragment

**DOI:** 10.1186/s12934-020-01487-x

**Published:** 2020-12-09

**Authors:** Wanjin Qiao, Yu Qiao, Fulu Liu, Yating Zhang, Ran Li, Zhenzhou Wu, Haijin Xu, Per Erik Joakim Saris, Mingqiang Qiao

**Affiliations:** 1 Key Laboratory of Molecular Microbiology and Technology, Ministry of Education, College of Life Sciences, Nankai University, No.94 Weijin Road, Nankai District, Tianjin, 300071 China; 2 Department of Microbiology, Faculty of Agriculture and Forestry, University of Helsinki, Helsinki, Finland; 3 State Key Laboratory of Medicinal Chemical Biology & Tianjin Key Laboratory of Protein Sciences, College of Life Sciences, Nankai University, Tianjin, China

**Keywords:** *Lactococcus lactis*, Prophage-related fragment, Genome editing, Multi-stress tolerance, Nisin immunity, Nisin yield, TMT quantitative proteomics

## Abstract

**Background:**

In bioengineering, growth of microorganisms is limited because of environmental and industrial stresses during fermentation. This study aimed to construct a nisin-producing chassis *Lactococcus lactis* strain with genome-streamlined, low metabolic burden, and multi-stress tolerance characteristics.

**Results:**

The Cre-*loxP* recombination system was applied to reduce the genome and obtain the target chassis strain. A prophage-related fragment (PRF; 19,739 bp) in the *L. lactis* N8 genome was deleted, and the mutant strain *L. lactis* N8-1 was chosen for multi-stress tolerance studies. Nisin immunity of *L. lactis* N8-1 was increased to 6500 IU/mL, which was 44.44% higher than that of the wild-type *L. lactis* N8 (4500 IU/mL). The survival rates of *L. lactis* N8-1 treated with lysozyme for 2 h and lactic acid for 1 h were 1000- and 10,000-fold higher than that of the wild-type strain, respectively. At 39 ℃, the *L. lactis* N8-1 could still maintain its growth, whereas the growth of the wild-type strain dramatically dropped. Scanning electron microscopy showed that the cell wall integrity of *L. lactis* N8-1 was well maintained after lysozyme treatment. Tandem mass tags labeled quantitative proteomics revealed that 33 and 9 proteins were significantly upregulated and downregulated, respectively, in *L. lactis* N8-1. These differential proteins were involved in carbohydrate and energy transport/metabolism, biosynthesis of cell wall and cell surface proteins.

**Conclusions:**

PRF deletion was proven to be an efficient strategy to achieve multi-stress tolerance and nisin immunity in *L. lactis*, thereby providing a new perspective for industrially obtaining engineered strains with multi-stress tolerance and expanding the application of lactic acid bacteria in biotechnology and synthetic biology. Besides, the importance of PRF, which can confer vital phenotypes to bacteria, was established.

## Background

The microbial cell factory, which mainly involves microorganisms to produce organic acids, chemicals, and antimicrobial peptides, is used for various purposes in industrial biotechnology [1]. Lactic acid bacteria (LAB) are commonly used as microbial cell factories [2] and *Lactococcus lactis* (*L. lactis*) is particularly employed to produce the food additive (E234) nisin. However, microorganisms often encounter environmental stress, including oxidative, acid, and heat stresses [3]. Moreover, nisin producers have to tolerate the stress caused by the antibacterial nisin. To date, only limited studies have reported on the construction of microbial chassis *L. lactis* strains to enhance multi-stress tolerance, reduce metabolic burden, and boost desirable product fermentation. Therefore, there is an urgent need to engineer LAB in order to adapt to extreme environmental pressures and accomplish normal growth and high production of valuable compounds [4].

In recent years, researchers have adapted strains with stress tolerance through different strategies, e.g., random mutagenesis, global transcription machinery engineering (gTME), global regulator overexpression and genome editing. Random mutagenesis has been extensively used to improve the acid tolerance of microbial cells. The global regulator Sigma D factor (RpoD) of *Escherichia coli* (*E. coli*) had been tailored by mutagenesis to achieve enhanced acid resistance, and the best mutant exhibited much higher growth rate than the control (0.22 h^−1^ vs 0.15 h^−1^) at pH 3.17 [5]. The ethanol tolerance of *Saccharomyces cerevisiae* (*S. cerevisiae*) had been increased after gTME by reprogramming gene transcription [6]. The survival rate of *E. coli* had been noted to increase by 10- to 100-fold at pH 2.5 by overexpressing the global regulator H-NS [7]. Li et al. overexpressed sHSP20 in *E. coli* BL21 cells and increased its survival period at 50 °C by almost 2 h [8], while deletion of *ADY2* improved the growth of *S. cerevisiae* under acetic acid, ethanol, and hydrogen peroxide stresses [9].

The two major methods to improve stress tolerance of LAB are heterologous expression [10] and endogenous overexpression [11]. In addition, genome shuffling has also been applied to improve acid tolerance and volumetric productivity of an industrial strain *Lactobacillus rhamnosus* ATCC 11443 [12]. Besides, small RNA like sRNA-*s015* [13] has also been reported to be an important factor that affects stress tolerance. Recently, the resistance or survival rates of *L. lactis* have been improved by exposing the bacterial cells to transglutaminase [14], bacteriocin Lcn972 [15], and Tween 80 [16] during growth.

In general, deletion of non-essential genome fragments, such as prophages and transposons, could confer some advantages on bacterial strains [17], including enhanced growth, increased biomass, and higher level of proteins synthesis [18]. Besides, prophages can usually synthesize proteins like prophage lysin that affect the host cell wall/membrane [19], and cell wall/membrane is essential for maintaining cellular integrity and resisting environmental stress [20]. For instance, the structure of the cell wall/membrane of nisin-producing *L. lactis* can also affect its tolerance to nisin [21]. Therefore, deletion of prophage-related fragments (PRFs) might affect multi-stress tolerance, nisin immunity, and nisin production of LAB. However, studies on the multi-stress tolerance of LAB are limited. For example, in a previous study, a putatively prophage-deleted derivative of *L. lactis* UC509 was constructed and the type and integrity of the prophage were investigated [22]. Likewise, *L. lactis* IL1403 derivatives were constructed and all the PRFs were deleted; however, the functions of these prophages in the strain were not explored [23]. Furthermore, a chassis derivative of *L. lactis* NZ9000 was generated, which exhibited superior growth phenotype and higher heterologous protein synthesis, but did not present multi-stress tolerance [4]. It must be noted that the above-mentioned studies did not include nisin-producing strains. The level of nisin production by a nisin producer could be regulated by the producer’s immunity to nisin [24]. Thus, to ensure growth and efficient production, *L. lactis* N8 must have the ability to resist nisin inhibition. In general, the nisin immunity of a producer is determined by different mechanisms, namely, structure of cell wall/membrane [21], local pH at the outer surface of the cytoplasmic membrane [25], nisin-digesting enzyme [26], and nisin immunity genes [27].

Bacterial tolerance is a complex regulatory mechanism. Before the advancement of proteomics technology, there was no systematic research method to understand bacterial tolerance at the protein level. Two-dimensional gel electrophoresis (2-DE) is a traditional method to identify “upregulated” and “downregulated” proteins [28]. However, 2-DE has limitations such as low resolution and bias against membrane proteins. In recent years, mass spectrometry (MS) technology has been widely used for the analysis of complex protein mixtures, and MS-based proteins quantitative analysis has replaced 2-DE proteomics. When compared with transcriptomics gene expression studies, proteomics can directly measure the level of gene products in a specific state and can further characterize protein activity, interaction, and subcellular distribution [29]. Proteomics has been successfully applied in various fields, such as determination of protein composition of organelles, elucidation of protein–protein interactions, and large-scale mapping of protein phosphorylation in response to stimuli [30]. Tandem mass tags (TMT)-labeled MS for protein synthesis analysis has been widely used to study the tolerance mechanism of microorganisms [29].

In the present study, TMT-labeled proteomics technology was applied to trace the changes in the protein synthesis of mutant strains. Functional analysis of differentially synthetized proteins was performed, and the protein response mechanism was elucidated based on enrichment results. The mechanism of multi-stress tolerance of the strain was also elaborated at the proteome level, and the results obtained can provide a basis for subsequent research in related fields. A PRF was deleted from *L. lactis* N8 genome using the Cre*-loxP* recombination system [31], and the mutant *L. lactis* N8-1 outperformed the wild-type strain in several physiological traits assessed, including better stress tolerance and higher nisin yield in acidic fermentation. TMT quantitative proteomics showed that the upregulated proteins were mainly enriched in sugar metabolism and biosynthesis of cell wall and cell surface proteins. The findings of this study provide new strategies for developing industrial strains with increased multi-stress tolerance and a novel perspective for the acquisition of highly robust strains.

## Results

### Design and construction of *L. lactis* N8-1

**Fig. 1:**
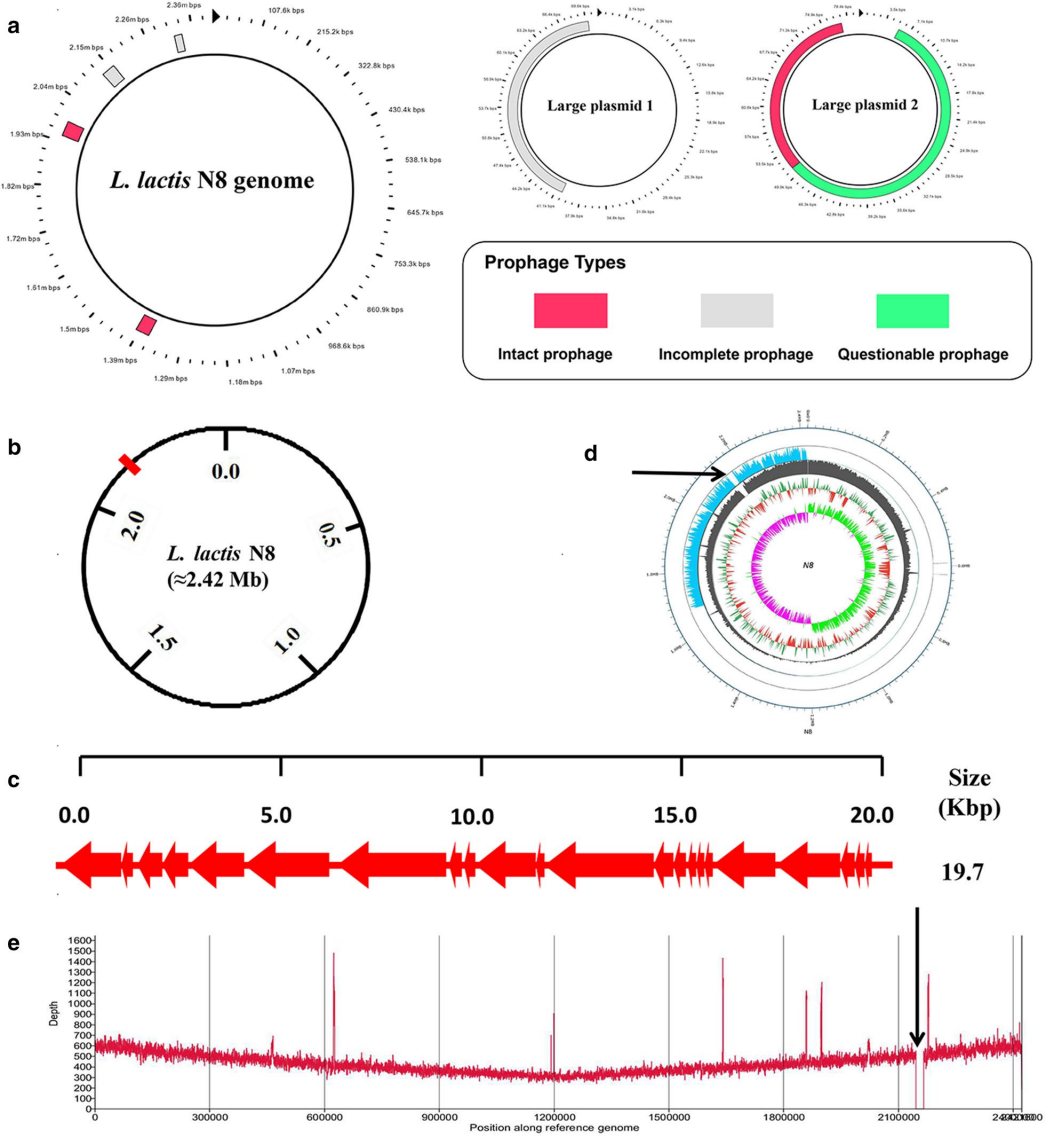
Circular map of *L. lactis* N8 chromosome and two large plasmids, and deletion of PRF DNA region in *L. lactis* N8. **a** Physical location of the seven PRFs. **b** Physical location of the deleted PRF in the genome. **c** Genetic organization of deleted PRF. **d** Resequencing results proved correct knockout. **e** Alignment of *L. lactis* N8 and *L. lactis* N8-1 genome sequences verified correct deletion

To construct the mutant strains, PHAST (PHAge Search Tool) was used for PRF prediction (http://phast.wishartlab.com/) [32]. The genome of *L. lactis* N8 contains seven PRFs (about 0.231 Mb), constituting 8.98% of the *L. lactis* N8 genome (about 2.57 Mb) (Fig. 1a). By using the Cre*-loxP* recombination system, one PRF was deleted. The location of the deleted PRF in *L. lactis* N8 is shown in Fig. 1b, and the genetic components of the deleted PRF are presented in Fig. 1c. The deleted PRF was 19.7 kb and contained 22 open reading frames. Among these genes, 14 have been characterized and one encodes phage integrase. A detailed description of the genes is provided in Additional file 1: Table S1. The deletion result was verified by PCR with confirmation primers and genome resequencing (Fig. 1d, e).

### Mutant strain showed increased nisin immunity

**Fig. 2:**
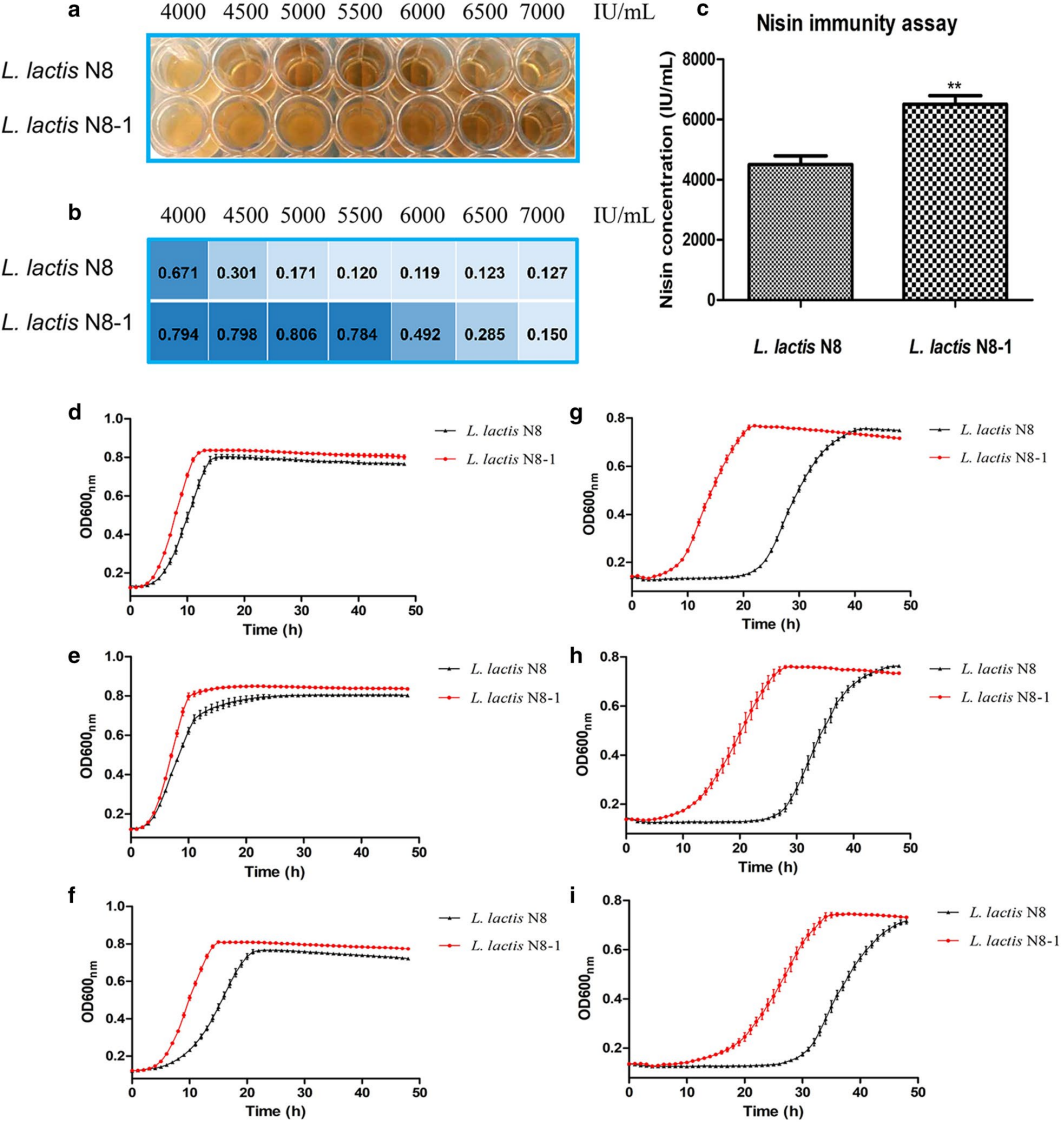
The nisin immunity and the growth profiles of *L. lactis* N8 and *L. lactis* N8-1 at GM17 medium supplemented with different concentrations of nisin. **a** 96-well plate gradient dilution cultures; **b** the result of the OD600 value of bacterial culture achieved by microplate reader; **c** nisin immunity of *L. lactis* N8 and *L. lactis* N8-1. **d** Growth curves at 4000 IU/mL nisin; **e** growth curves at 5000 IU/mL nisin; **f** growth curves at 6000 IU/mL nisin; **g** growth curves at 6500 IU/mL nisin; **h** growth curves at 7000 IU/mL nisin; **i** growth curves at 7500 IU/mL nisin

Analysis of the nisin immunity of the *L. lactis* N8-1 showed that there was significant difference between the mutant and wild-type strains in nisin immunity (Fig. 2a, b). *L. lactis* N8-1 presented 44.44% higher nisin resistance than *L. lactis* N8 (Fig. 2c). The growth curves of the wild-type and mutant strains were obtained under different concentrations of nisin, and revealed that there were no significant differences in growth between the mutant and wild-type strains at 4000 IU/mL (Fig. 2d), 5000 IU/mL (Fig. 2e), and 6000 IU/mL (Fig. 2f) nisin concentrations. These results indicated that the wild-type and mutant strains could grow in GM17 medium with high concentration of nisin [6500 IU/mL (Fig. 2g), 7000 IU/mL (Fig. 2h), and 7500 IU/mL (Fig. 2i)]; however, the wild-type strain started to grow was 15–20 h later than the mutant strain, suggesting that *L. lactis* N8-1 took less time to overcome the side effects of nisin and start to grow. These findings indicated that *L. lactis* N8-1 has better nisin immunity than *L. lactis* N8. Moreover, although nisin producers were not killed by high concentrations of nisin to a certain extent, their growth was still restrained.

### Mutant strain exhibited increased resistance to lysozyme

**Fig. 3:**
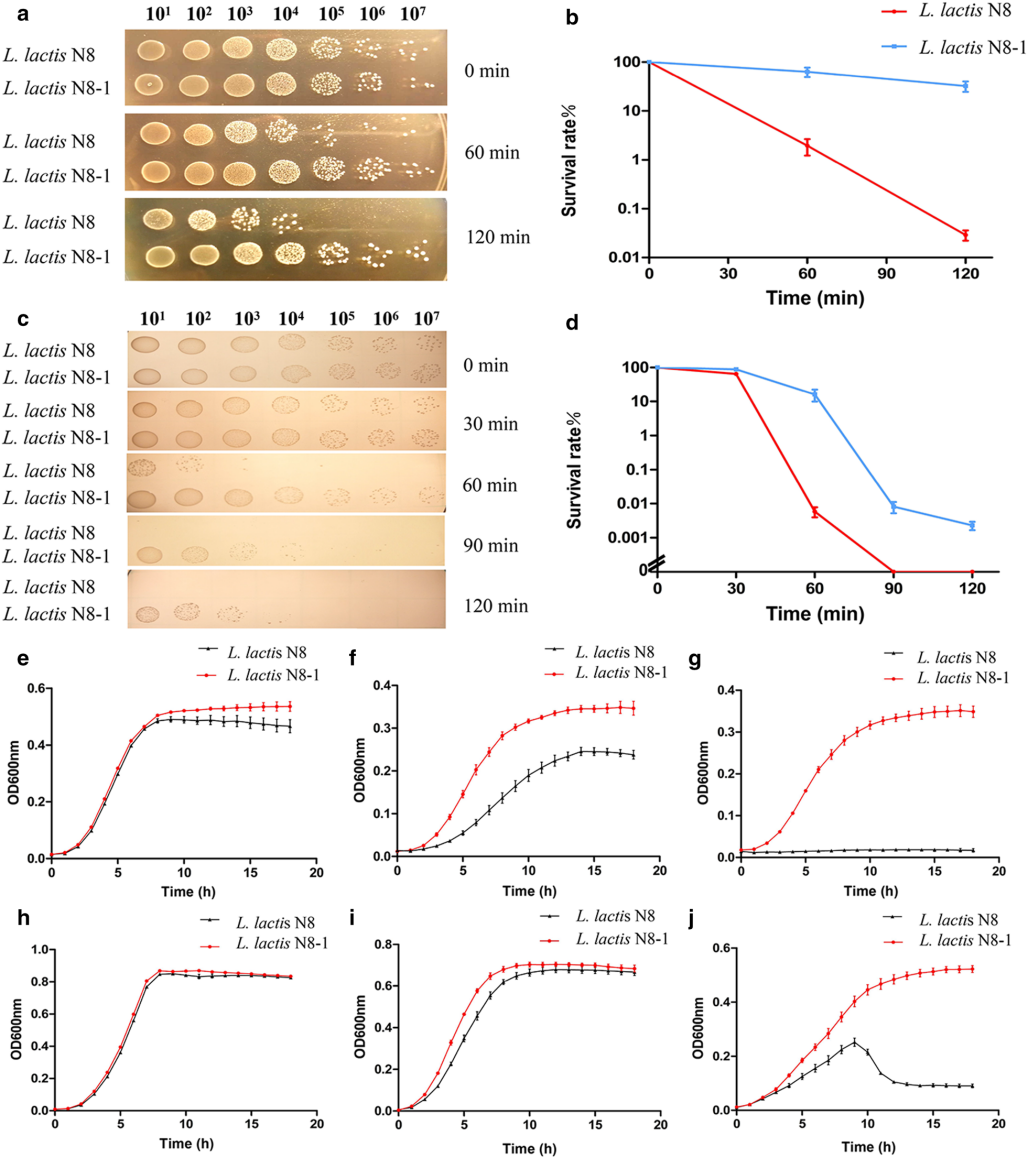
The survival rates and growth profiles of *L. lactis* N8 and *L. lactis* N8-1 under different conditions. **a** Gradient dilution drop plate experiment treated with lysozyme (10 mg/mL). **b** Survival rate curves treated with lysozyme (10 mg/mL). **c** Gradient dilution drop plate experiment treated with lactic acid [1.5% (v/v)]. **d** Survival rate curves treated with lactic acid [1.5% (v/v)]. **e** Growth curves at 30 ℃; **f** growth curves at 37 ℃; **g** growth curves at 39 ℃. **h** Growth curves under 0.24% (v/v) lactic acid; **i** growth curves under 0.32% (v/v) lactic acid; **j** growth curves under 0.40% (v/v) lactic acid

The lysozyme tolerance of *L. lactis* N8 and *L. lactis* N8-1 was evaluated by drop plate experiments. The survival rates of *L. lactis* N8 and *L. lactis* N8-1 after lysozyme treatment were determined to clarify the difference between both the strains. The *L. lactis* N8-1 exhibited higher survival rates after treated with lysozyme at various time points (Fig. 3a). After treatment for 60 min, *L. lactis* N8-1 exhibited 75.3-fold higher survival rate, when compared with *L. lactis* N8. Moreover, after treatment for 120 min, the survival rate of *L. lactis* N8-1 was markedly higher (1000-fold) than that of the control strain (Fig. 3b). These results demonstrated that loss of PRF conferred *L. lactis* N8-1 lysozyme tolerance.

### Mutant strain presented increased resistance to lactic acid

To determine lactic acid tolerance, the growth curves of *L. lactis* N8 and *L. lactis* N8-1 under different concentrations of lactic acid were acquired (Fig. 3). There was no significant difference between the OD600 of *L. lactis* N8 and *L. lactis* N8-1 when the concentration of lactic acid was 0.24% (v/v) (Fig. 3e). In contrast, when the concentration of lactic acid was increased to 0.32% (v/v), the OD600 of the mutant strain was 58% higher than that of the wild-type strain after 18 h of incubation (Fig. 3f). The mutant strain continued to grow, whereas the wild-type strain could hardly grow when the lactic acid concentration was increased to 0.40% (v/v) (Fig. 3g). Drop plate experiment showed that *L. lactis* N8-1 exhibited higher survival rates at various time points after being treated with lactic acid [1.5% (v/v)], and the survival rate of *L. lactis* N8-1 was significantly higher than that of *L. lactis* N8 (10,000-fold) after 60 min of treatment (Fig. 3c, d).

### Mutant strains reached higher cell density at high temperature

To determine the tolerance of the mutant strain to high temperature, the growth curves of *L. lactis* N8 and *L. lactis* N8-1 under different temperatures were acquired. There was no significant difference between the OD600 of *L. lactis* N8 and *L. lactis* N8-1 when the temperatures were 30 ℃ (Fig. 3h) and 37 ℃ (Fig. 3i). However, when the temperature was raised to 39 ℃, significant difference in the OD600 was observed between *L. lactis* N8 and *L. lactis* N8-1 (Fig. 3j). After 9 h, the growth of *L. lactis* N8 drastically decreased, while *L. lactis* N8-1 could still grow. The growth curves obtained at 39 ℃ revealed abnormal *L. lactis* N8 growth, indicating that high temperature had disrupted its normal growth; in contrast, the growth of *L. lactis* N8-1 was still normal.

### Mutant strains showed higher utilization of several carbon sources

Extensive fermentation phenotype analyses of *L. lactis* N8-1 and *L. lactis* N8 were conducted using the phenotype microarrays to further understand the physiological differences between the wild-type and mutant strains. Additional file 1: Table S2 summarizes the comparisons of substrates consumed by *L. lactis* N8-1 and *L. lactis* N8. The results showed that the mutant strain could efficiently metabolize some substrates, including α-d-glucose, d-mannose, sucrose, and *N*-acelyl-d-glucosamine, which are associated with carbon source metabolism and cell wall biosynthesis. The mutant *L. lactis* N8-1 exhibited distinct properties with respect to metabolism of four carbon sources, when compared with the wild-type strain.

### Scanning electron microscopy of cells after lysozyme treatment

**Fig. 4:**
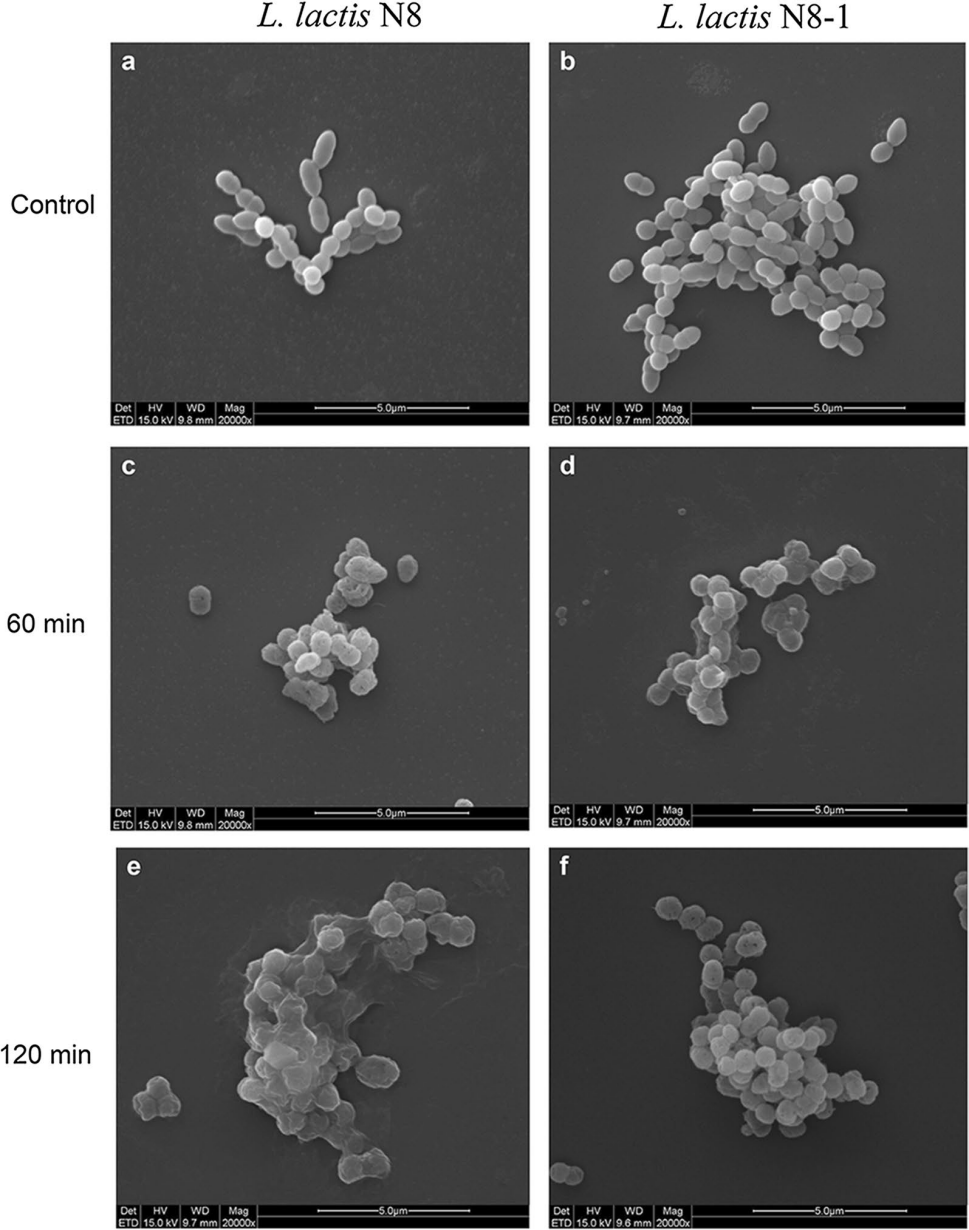
Scanning electron microscopy (SEM) of *L. lactis* N8 and *L. lactis* N8-1 treated with lysozyme (17.5 mg/mL)

To determine the causes of multi-stress tolerance of the mutant *L. lactis* N8-1, the morphology and cell integrity of *L. lactis* N8 and *L. lactis* N8-1 after lysozyme treatment were examined by scanning electron microscopy (SEM). As shown in Fig. 4, following lysozyme treatment (17.5 mg/mL) for 60 min, the cell wall of *L. lactis* N8-1 maintained better integrity, whereas that of *L. lactis* N8 presented obvious holes. After 120 min of lysozyme treatment, the cell wall of *L. lactis* N8-1 was damaged, while that of *L. lactis* N8 was almost completely degraded. These results (from a micro-perspective) revealed that the cell wall of the mutant *L. lactis* N8-1 was more resistant to lysozyme, and confirmed that *L. lactis* N8-1 exhibited better robustness.

### Mutant strains achieved higher nisin yield under acidification conditions

**Fig. 5:**
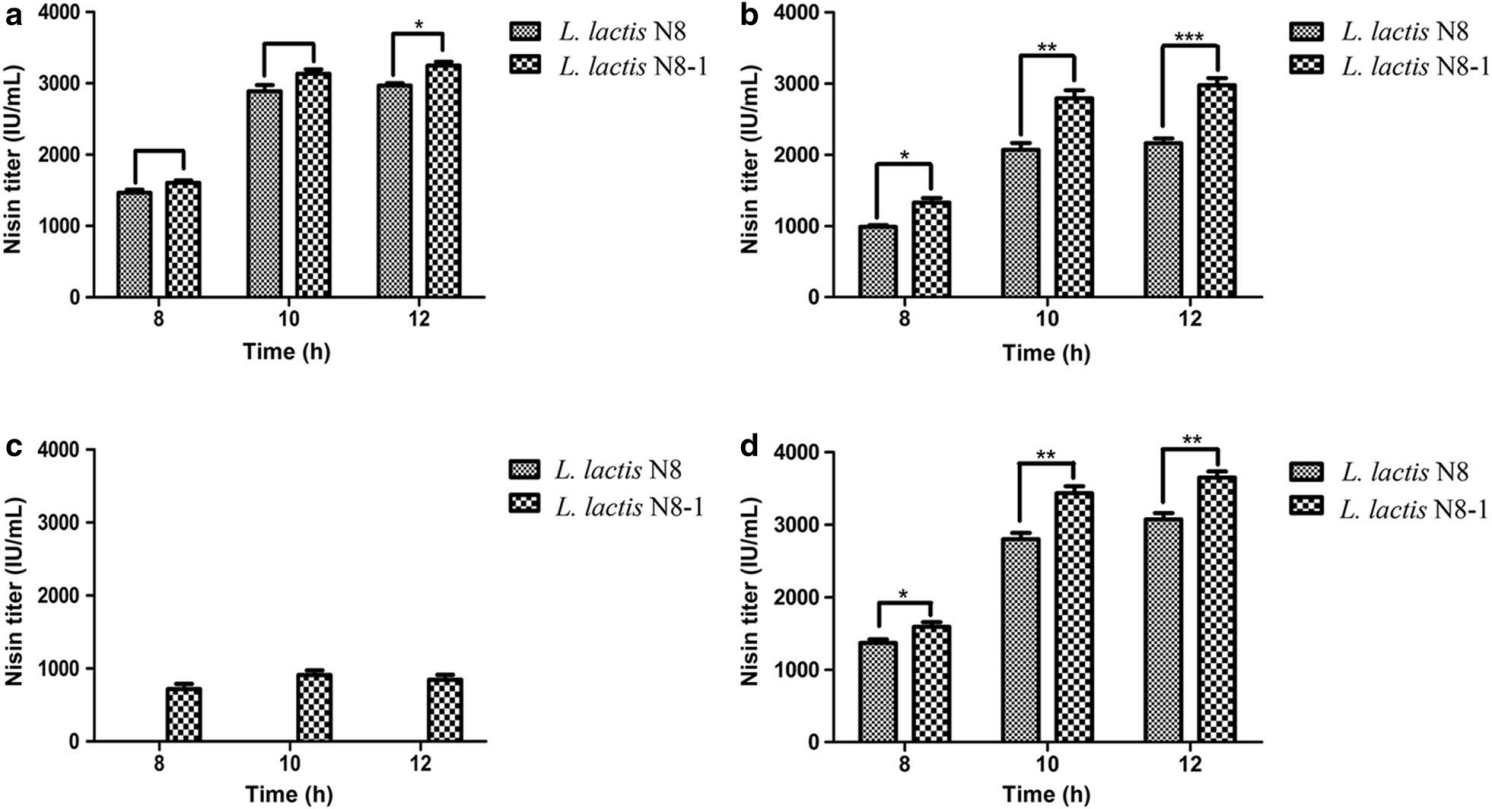
Comparison of the nisin yield of *L. lactis* N8 and *L. lactis* N8-1. **a** Nisin yield at pH 6.0; **b** Nisin yield at pH 5.5; **c** Nisin yield at pH 5.0; **d** Nisin yield of the wild-type and mutant strains by overexpressing *nisZ*

To obtain strains with superior nisin immunity and nisin production, we constructed engineered strains to obtain a high nisin yield chassis. However, after overexpressing the nisin immunity genes, *nisFEG*, *nisIFEG*, and *nisRKFEG*, in *L. lactis* N8-1, we were unable to obtain mutant strains with higher nisin immunity characteristics (Additional file 1: Table S3). As strains used in industrial production are known to endure acid stress, we used lactic acid to adjust the pH of the medium for constant-pH fermentation. As shown in Fig. 5a, there was no obvious difference in nisin production between *L. lactis* N8 and *L. lactis* N8-1 when the pH of the medium was 6.0. However, when the medium pH was adjusted to 5.5, the nisin yield of *L. lactis* N8-1 was 36.29% higher than that of *L. lactis* N8 at 12 h (Fig. 5b). When the pH of the medium was adjusted to 5.0, *L. lactis* N8 had limited growth with no increased nisin production, while the growth of *L. lactis* N8-1 was significantly restrained, but presented considerable nisin production (Fig. 5c). Subsequently, we overexpressed *nisZ* in *L. lactis* N8-1 and *L. lactis* N8 and found that the nisin yield of *L. lactis* N8-1 was 22.57% and 18.79% higher than those of the wild-type strain at 10 and 12 h, respectively. (Fig. 5d). These results indicated that *L. lactis* N8-1 is more advantageous than *L. lactis* N8 as a microbial cell factory with higher nisin yield and nisin immunity.

### TMT quantitative proteomics analysis and transcription verification

**Fig. 6:**
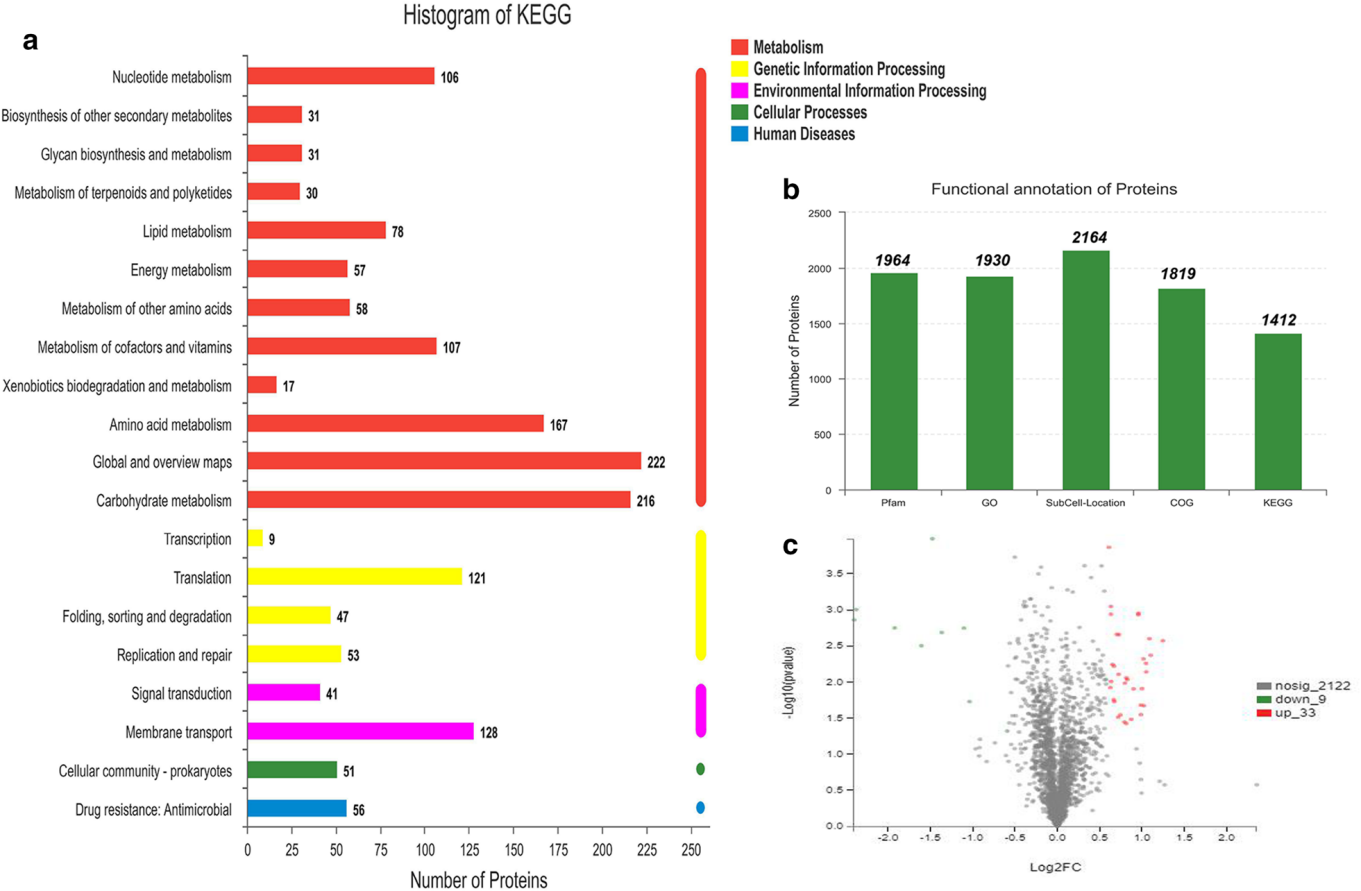
Overview of proteomics parameters. **a** Comparing the identified proteins with the KEGG database and all proteins are classified into 20 metabolic pathways. All metabolic pathways belong to five categories: red bars represent *Metabolism*, yellow bars represent *Genetic Information Processing*, purple bars represent *Environmental Information Processing*, green bars represent *Cellular Processes*, and blue bars represent *Human Diseases.* **b** Proteomic functional annotation against (Pfam, GO, Uniprot, COG, and KEGG) databases. **c** Volcano map of significantly upregulated and downregulated proteins in *L. lactis* N8-1 compared with *L. lactis* N8

The mutant strain *L*. *lactis* N8-1 with improved nisin immunity and multi-stress tolerance (lysozyme, lactic acid, and high-temperature tolerance) was constructed. Based on the results of TMT quantitative proteomics, a total of 2164 proteins were detected. Different databases were used to annotate proteins, while KEGG database was used to classify the proteins (Fig. 6a, b). We found that 33 proteins were significantly upregulated and nine proteins were significantly downregulated (> 1.5-fold change with *P* < 0.05) in *L. lactis* N8-1, when compared with those in *L. lactis* N8 (Fig. 6c). The upregulated proteins and downregulated proteins (> 1.2-fold change with a *P* < 0.05) are listed in Additional file 1: Tables S4, S5, respectively. KEGG functional enrichment analysis (*L. lactis* N8-1 vs *L. lactis* N8) showed that the upregulated proteins were mainly related to sugar metabolism and amino acid biosynthesis/metabolism (Additional file 1: Fig. S1a). In contrast, the downregulated proteins were mainly related to ribosome biosynthesis (Additional file 1: Fig. S1b). The gene ontology (GO) function annotations for all proteins (and upregulated and downregulated proteins) are shown in Additional file 1: Fig. S2. Protein subcellular localization analysis showed that the upregulated proteins were mainly sub-located in the cytoplasm and extracellular (43 proteins), whereas the downregulated proteins were mainly sub-located in the cytoplasm (34 proteins) (Additional file 1: Fig. S3). Subsequently, several genes with higher and lower expression levels were examined by real-time quantitative PCR (RT-qPCR) to verify the proteomics data. When compared with *L. lactis* N8, the expression levels of genes *rpsN, rplR, csc2B, csc2C, pi339, pp423, butA, butB, arcA, arcB, galM, galK and lacZ* were altered in *L. lactis* N8-1 (about 0.37-, 0.32-, 2.55-, 3.17-, 3.00-, 3.79-, 3.52-, 2.20-, 3.51-, 2.37-, 4.49-, 5.60-, and 2.55-fold, respectively) (Additional file 1: Fig. S4), which were in agreement with the results of proteomics assay.

## Discussion

In laboratory or industrial fermentation process, microbial strains are generally believed to endure many harsh conditions, including oxidation, heating and cooling, acid, high osmolarity/dehydration, and starvation [33]. Therefore, understanding of the stress response behavior of *L. lactis* is of crucial significance for expanding its application in industrial fermentation. In recent years, an increasing number of studies have confirmed that *L. lactis* can survive by activating specific protection mechanisms in response to environmental pressure [34]. To tolerate heat shock, *L. lactis* regulates the folding and maturation of new or denatured proteins through the synthesis of heat shock proteins (HSPs). Genetic analyses have confirmed the presence of the conserved HSPs in *L. lactis*, including DnaK, DnaJ, GroEL, GroES, and GrpE [33]. Under low temperature pressure, *E. coli* can prevent the formation of secondary structures in RNA molecules and stimulate translation efficiency by regulating the cold shock proteins (CSPs), CspA [35]. Researchers have identified conserved CSPs (CspA, CspB, CspC, CspD, and CspE) in *L. lactis* and proved the important role of these CSPs under cold shock conditions [36]. Under low pH condition, LAB mainly employ two mechanisms to respond to stimuli. The primary mechanism for the control of intracellular pH is F_0_F_1_ ATPase that translocates protons to the environment at the expense of ATP. Both the expression level and activity of this protein complex have been noted to increase at low pH [37]. The second mechanism in response to low pH is the arginine deiminase pathway that allows *L. lactis* to neutralize its environment through NH_3_ production [38]. To overcome oxidative stress, some *L. lactis* strains have been reported to accumulate glutathione and possibly utilize glutathione to remove O_2_^−^ [39, 40]. Conversely, the expression of *recA* has been noted to be induced in aerated cultures and a *recA* mutant *L. lactis* has been found to be highly sensitive to aeration, indicating that *recA* gene clearly plays a role in oxidative stress [41]. In the past decades, numerous studies have been conducted to improve strain stress tolerance, and heterologous expression and overexpression in LAB have gained much research attention. Sugimoto et al. constructed multi-stress tolerant (3% NaCl, 5% ethanol, and 0.5% lactic acid) *L. lactis* NZ9000 by heterologous expression of *dnaK* gene from *E. coli* [42]. Hagi et al. proved that the heterologous expression of *crtNM* in *L. lactis* MG1363 led to the increased H_2_O_2_, low pH, 20% bile acid, and 12 mg/mL lysozyme tolerance [43]. Furthermore, Desmond et al. improved stress tolerance by overexpressing *GroESL* in *Lactobacillus paracasei* NFBC 338 [solvent tolerance, the ability to grow in the presence of butanol (0.5% v/v) for 5 h] [11], while Zhu et al. overexpressed ABC transporters in *L. lactis* NZ9000 and obtained enhanced acid-stress tolerance derivatives [44]. The common strategy that had been applied in these studies is the so-called “plus” method to achieve high synthesis level of the target proteins. In contrast, large non-essential fragments, such as prophages, transposons, and genomic islands, as exogenously inserted chromosomal sequence, are not necessary for the host cells. It has been reported that deletion of large non-essential fragments produced the phenotypes with reduced metabolic burden, faster growth, and increased biomass [4]. Therefore, the “minus” method could be considered as a promising approach.

In the present study, after deleting a PRF, the mutant strain *L. lactis* N8-1 did not show growth deficiency, when compared with the wild-type strain. Besides, the genome of the mutant strain *L. lactis* N8-1 was streamlined, which is beneficial for the construction of engineered LAB microbial cell factories. The mutant *L. lactis* N8-1 exhibited significantly improved nisin immunity, which may provide a good basis for achieving high production of nisin. Proteomics analysis showed that the synthesis of NisI and NisFEG proteins was not increased in the mutant strain. Subsequently, we tried to overexpress the genes *nisFEG, nisIFEG* and *nisRKFEG* in *L. lactis* N8-1; however, no strains with significantly higher nisin immunity were obtained, indicating that beyond a certain range, nisin immunity tends to be under a global and complex regulation.

Besides the substantial increment in nisin immunity, the mutant strains also presented heat, lactic acid, and lysozyme resistances. We speculated that the improved multi-stress tolerance of the mutant strains was owing to the loss of PRF, resulting in remodeling of the cell wall/membrane and changes in carbon source metabolism. Previous studies have shown that the structure of the cell wall/membrane affects the nisin tolerance of LAB [45], and cell wall, as the first barrier of bacteria, is essential for the strain’s stress tolerance [20]. Similarly, in the present study, the synthesis levels of enzymes related to cell wall/membrane biosynthesis and degradation in *L. lactis* N8-1 were noted to be altered. Thus, combined with the results of lysozyme treatment experiment and SEM, these evidences demonstrated that deletion of PRF resulted in remodeling of the mutant’s cell wall/membrane. Based on the possible pleiotropic changes caused by PRF knockout, the proteins that led to stress tolerance in the mutant strain could be discussed in the following three groups: (1) carbohydrate and energy transport/metabolism; (2) synthesis of peptidoglycan (PG), cell wall, and cell surface proteins; and (3) others (Table 1). The findings of this study could help us to further understand the possible mechanism of stress tolerance of *L. lactis*, and lay a foundation to explore the deeper and detailed underlying reasons in the future as well as provide significant guidelines for the construction of robust microbial cell factories.

**Fig. 7:**
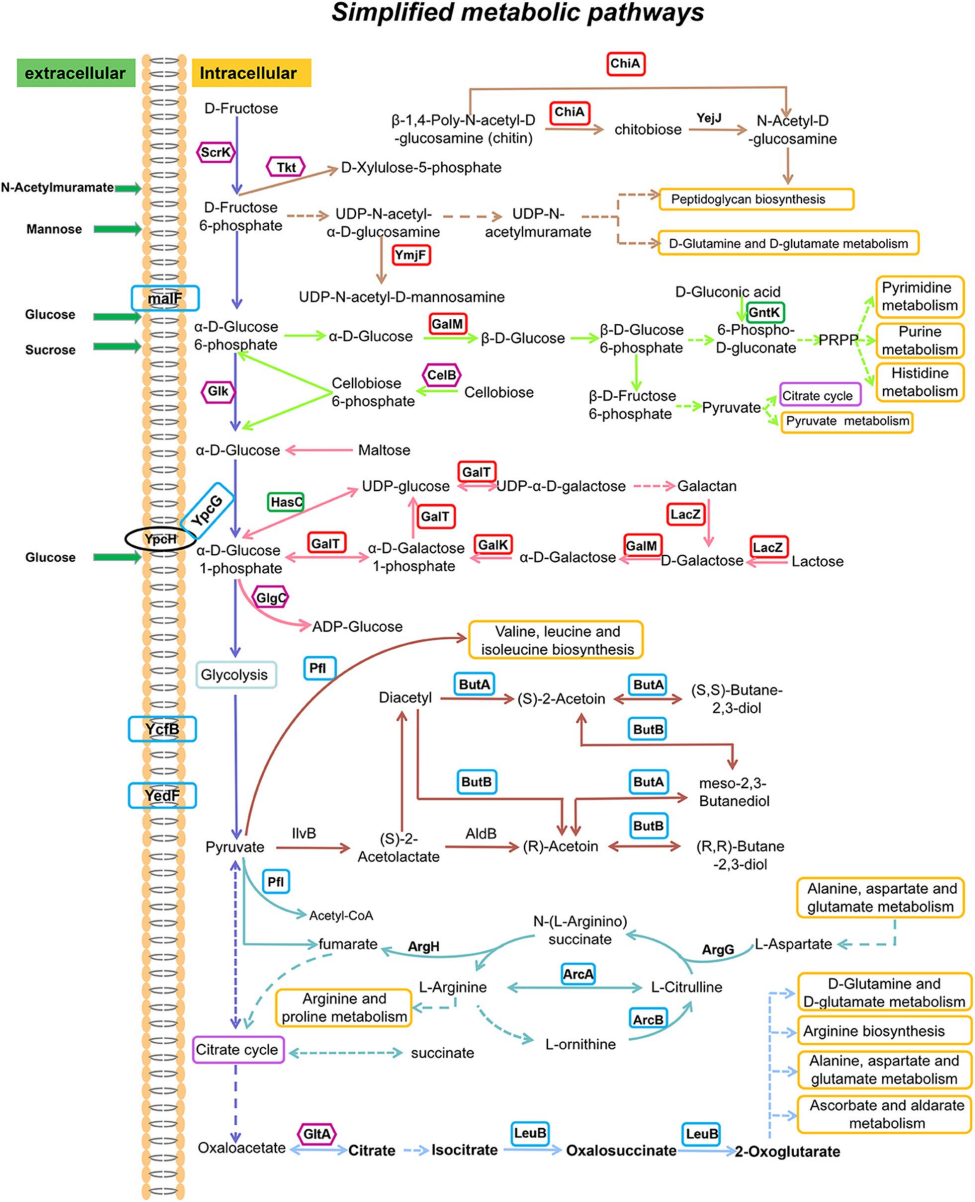
Simplified metabolic pathways of *L. lactis*. The significantly upregulated (> 1.5-fold change with *P* < 0.05) proteins are represented by red frames; upregulated (1.2–1.5-fold change with *P* < 0.05) proteins are represented by blue frames; upregulated (> 1.2-fold change with *P* > 0.05) proteins are represented by purple frames; downregulated (> 1.2-fold change with *P* < 0.05) proteins are represented by green frames

1. Carbohydrate and energy transport/metabolism
  Carbohydrate and energy transport/metabolism are the basic life activities of bacteria. Changes in the synthesis of enzymes that catalyze these processes often produce a huge impact on the bacterial phenotype. The proteomics results of the present study indicated that the protein synthesis levels of GalM (aldose 1-epimerase), GalK (galactokinase), and GalT (galactose-1-phosphate uridylyltransferase), which belong to galactose metabolism pathway, were significantly upregulated in *L. lactis* N8-1. The upregulation of these proteins allowed the metabolic flow to α-d-glucose 1-phosphate, which can be used as an intermediate substrate to participate in glycolysis, pentose and glucuronate interconversions, etc. As the intermediate product of glycolysis, pyruvate participates in sugar metabolism, amino acid metabolism, citrate cycle, and fatty acid metabolism. Previous studies have shown that the *gal* operon plays a role in the nisin tolerance, and that *L. lactis* NZ9000△*galAMK* was twice as sensitive to nisin as its parent strain [46]. However, the contributions of the mechanisms of these transport systems to stress resistance remain to be elucidated. It has been speculated that sugar metabolism intermediates in cell wall biosynthesis or PTS systems might contribute to energy for stress tolerance mechanism [46]. In the present study, the synthesis level of LacZ (β-galactosidase), LacA (galactoside *O*-acetyltransferase), and LacS (lactose permease) was significantly upregulated in *L. lactis* N8-1. LacZ catalyzes the hydrolysis of terminal non-reducing β-d-galactose residues in β-d-galactosides, while LacA and LacS are sugar transport proteins. The proteins of this family are major facilitators of membrane transport and may contribute to energy supply in stress tolerance mechanism. Overall, all these mechanisms above could contribute to the ability of the mutant strains to completely utilize carbon source and may provide a basis for the strain’s multi-stress tolerance.
2. Biosynthesis of PG, cell wall and cell surface proteins
  Cell wall is essential for stress tolerance in bacteria. Under constantly changing living conditions, enzymes that are involved in PG synthesis and degradation can regulate the dynamic balance of the strain’s cell wall. In *L. lactis* N8-1, YmjE (glycosyl transferase) and YmjF (UDP-*N*-acetyl glucosamine 2-epimerase), which are essential for the biosynthesis of cell wall surface polysaccharides [47], were significantly upregulated. Cell wall surface polysaccharides have been confirmed to contribute to nisin immunity of LAB [25]. In *L. lactis* N8-1, synthesis of cell surface proteins, Csc2A, Csc2B, and Csc2C, were significantly upregulated. Siezen et al. speculated that the proteins from cell surface protein complexes may play an important role in carbon source acquisition of bacteria [48]. The gene from *cscABCD* gene cluster encodes WxL-domain-containing cell surface proteins and LPxTG-anchored cell surface proteins. The WxL motif confers protien cell surface localization function, and this region is the cell wall-binding domain of Gram-positive bacteria and may interact with PG [49]. Besides, the synthesis of YbeF (LPxTG collagen binding domain-containing protein) in *L. lactis* N8-1 was significantly increased. Pieterse et al. showed that the synthesis of LPxTG-anchored cell surface protein was significantly improved after treatment with lactic acid [50], suggesting that LPxTG-anchored cell surface protein contributes to lactic acid resistance. As these two proteins are anchored on the cell wall/membrane to some extent, we proposed that the cell surface proteins are likely to participate in the stress tolerance of the strain and further protect it. The synthesis and degradation of cell wall is a precise regulation process and maintains certain balance [51]. We speculated that the deletion of PRF could promote remodeling of cell wall, which may eventually make the cell wall become denser or form a protective structure, ultimately providing multi-stress tolerances.
3. Others
  In addition to the above-mentioned changes, the synthesis levels of 9 proteins belonging to a complete prophage were significantly upregulated in the mutant strain. Besides, the synthesis of several other bacteriophage infection proteins was also upregulated. A previous study had demonstrated that *L. lactis* IL1403 acquired stress tolerance (antimicrobial, heme) after deleting all the prophages [23]; however, the study did not clarify the changes in the strain at the transcriptional or translational level; therefore, it is difficult to determine the prophage that conferred stress tolerance to the strain. In the present study, comparison of the PRF deleted from *L. lactis* N8 with all the PRFs of *L. lactis* IL1403 revealed no correlation, indicating that deletion of different types of PRF confers diverse characteristics to the strain. However, protein synthesis of a prophage that still remained on the mutant’s genome was increased, suggesting that the absence of a certain type of PRF could affect the protein synthesis of other prophages even in the lysogenic state. It is presumed that recognition and competition mechanisms of the prophages could ultimately affect the phenotype of the strain itself.
  Thus, it can be concluded that the acquired multi-stress tolerance of the mutant strain *L. lactis* N8-1 is a very complex trait. Figure 7 summarizes the simplified metabolic pathways. Many proteins possibly involved in stress tolerance were detected and two major mechanisms of acquiring stress tolerance could hypothesized. The first mechanism of acquiring stress tolerance could possibly be through changes in protein synthesis of prophage, with lysophage indirectly contributing to the stress tolerance of the host bacteria; however, detailed reason still needs to be explored. The second and major mechanism could be the changes in the cell wall structure. The cell wall of the *L. lactis* N8-1 might have probably been altered in two ways: becoming denser (LacS, YmjE, YmjF) and getting thicker (Csc2A, Csc2B, Csc2C).

**Table 1:** Partial upregulated proteins found in proteomic analysis (*L. lactis* N8-1 vs *L. lactis* N8)

Protein name	Description	fc (N8-1/N8)	*P*-value (N8-1/N8)
Carbohydrate and energy transport/metabolism			
GalM	Aldose 1-epimerase	1.8319	0.0328
GalK	Galactokinase	1.9828	0.0283
GalT	Galactose-1-phosphate uridylyltransferase	1.6774	0.0286
LacA	Galactoside *O*-acetyltransferase	1.6464	0.0307
LacZ	Beta-galactosidase	1.7593	0.0376
LacS	Lactose and galactose permease GPH translocator family	1.5916	0.0060
Biosynthesis/degradation of PG, cell wall and cell surface proteins			
YmjE	Glycosyl transferase	1.5957	0.0187
YmjF	UDP-N-acetylglucosamine 2-epimerase	2	0.0123
YuaE	Aspartate protease	1.5713	0.0057
ChiA	Chitinase	1.6291	0.0021
YqcD	WxL domain-containing protein	2.0262	0.0047
Csc2A	Cell surface protein	1.5525	0.0011
Csc2B	WxL domain-containing cell surface protein	2.1488	0.0042
Csc2C	WxL domain-containing cell surface protein	1.8678	0.0124
YqbH	Transcriptional regulator	1.7327	0.0358
YbeF	Collagen binding domain-containing protein	2.3745	0.0026
Others (phage-related proteins)			
Pip	Phage infection protein	1.9826	0.0209
NA	Phage antirepressor	1.5872	0.0176
Pp261	Uncharacterized protein	1.5545	0.0098
Pi339	Prophage pi3 protein 39	1.7602	0.0087
Pp423	Phage transcriptional regulator, ArpU family	1.9412	0.0011
Phi3396	Phage major capsid protein	1.6566	0.0022
NA	Phage tail protein	1.9425	0.0011
NA	Prophage protein	1.6534	0.0078
Pi308	Prophage pi3 protein 34	1.7759	0.0092
YjaE	YhgE/Pip (phage infection protein) domain-containing protein	2.0323	0.0212

## Conclusions

This study demonstrated that bacterial cells employ diverse mechanisms to defend themselves against multiple stresses. To the best of our knowledge, this is the first report to improve the multi-stress tolerance and nisin immunity of *L. lactis* by deleting PRF. TMT quantitative proteomics was proven to be an efficient technique to systematically and comprehensively elucidate the possible mechanisms of multi-stress tolerance of PRF knockout strain, which provides a new strategy for industrially obtaining robust *L. lactis* microbial cell factories with superior tolerance and higher nisin immunity.

## Methods

### Bacterial strains, plasmids and growth conditions

The strains and plasmids used in this study are listed in Table 2. *L. lactis* N8 (wild-type nisin Z producer) and its derivatives were cultured at 30 ℃ without agitation in GM17 medium [M17 broth supplemented with 0.5% (w/v) glucose]. *E. coli* DH5α cells were grown aerobically at 37 ℃ in Luria–Bertani (LB) broth (1% tryptone, 0.5% yeast extract, and 1% NaCl) and used as cloning host. *Micrococcus luteus* NCIB 8166 was cultured aerobically in LB Broth at 37 ℃ and used as nisin sensitive indicator strain to detect the antibacterial activity of nisin. *L. lactis* NZ9000 was used as intermediate cloning host for the pNZ8048/pLEB124 plasmids construction, and it was cultured at 30 ℃ without agitation in GM17 medium. While pNZ8048/pLEB124 was used to construct the expression vector, pNZ5319 was employed to construct the knock-out vector through Cre*-loxP* gene recombination system. Antibiotics were used when needed: 150 and 5 μg/mL erythromycin for *E. coli* and *L. lactis,* respectively*;* 15 and 5 μg/mL chloramphenicol for *E. coli* and *L. lactis,* respectively; 100 μg/mL kanamycin for *E. coli*; and 20 μg/mL ampicillin for *L. lactis.*

**Table 2:** Bacterial strains and plasmids utilized in this study

Strains or plasmids	Relevant descriptions	Reference
Strains		
*E. coli* DH5α	Cloning host; F-φ80*lacZ*△M15*endA1 recA1 endA1 hsdR17* (rK-mK+) *supE44 thi-1 gyrA 96 relA1* △(*lacZYA-argF*)*U169 deoR* λ-	[58]
*E. coli* DH5α-up	Cm^r^, Em^r^, *E. coli* DH5α derivative containing the whole plasmid pNZ5319-up	This study
*E. coli* DH5α-up-down	Cm^r^, Em^r^, *E. coli* DH5α derivative containing the whole plasmid pNZ5319-up-down	This study
*Micrococcus luteus* NCIB 8166	Indicator strains for Nisin agar gel diffusion assay	[59]
*L. lactis* NZ9000	MG1363 *pepN::nisRK*	[60]
*L. lactis* N8	Wild-type (WT) Nisin Z producer	[27]
*L. lactis* N8-up-down	Cm^r^, Em^r^, *L. lactis* N8 derivative containing the whole plasmid pNZ5319-up-down (single cross-over)	This study
*L. lactis* N8-c*at*	Cm^r^,*L. lactis* N8 derivative containing the a *lox66*-P32-*cat*-*lox71* replacement of PRF (double cross-over)	This study
*L. lactis* N8-1	Mutant. The PRF deletion from *L. lactis* N8	This study
*L. lactis* N8-vector1	Cm^r^, *L. lactis* N8 derivative containing pNZ8048	This study
*L. lactis* N8-*nisZ*	Cm^r^, *L. lactis* N8 derivative containing pNZ8048-*nisZ*	This study
*L. lactis* N8-1-vector1	Cm^r^, *L. lactis* N8-1 derivative containing pNZ8048	This study
*L. lactis* N8-1-*nisZ*	Cm^r^, *L. lactis* N8-1 derivative containing pNZ8048-*nisZ*	This study
*L. lactis* N8-vector2	Em^r^, *L. lactis* N8 derivative containing pLEB124	This study
*L. lactis* N8-*nisFEG*	Em^r^, *L. lactis* N8 derivative containing pLEB672	This study
*L. lactis* N8-*nisIFEG*	Em^r^, *L. lactis* N8 derivative containing pLEB124-*nisIFEG*	This study
*L. lactis* N8-*nisRKFEG*	Em^r^, *L. lactis* N8 derivative containing pLEB674	This study
*L. lactis* N8-1-vector2	Em^r^, *L. lactis* N8-1 derivative containing pLEB124	This study
*L. lactis* N8-1-*nisFEG*	Em^r^, *L. lactis* N8-1 derivative containing pLEB672	This study
*L. lactis* N8-1-*nisIFEG*	Em^r^, *L. lactis* N8-1 derivative containing pLEB124-*nisIFEG*	This study
*L. lactis* N8-1-*nisRKFEG*	Em^r^, *L. lactis* N8-1 derivative containing pLEB674	This study
Plasmids		
pNZ5319	Cm^r^, Em^r^, used as knock-out vector	[31]
pNZ5319-up	Cm^r^, Em^r^, upstream homology arm amplified from *L. lactis* N8 genome cloned into pNZ5319	This study
pNZ5319-up-down	Cm^r^, Em^r^, upstream and downstream homology arm amplified from *L. lactis* N8 genome cloned into pNZ5319	This study
pNZTS-Cre	Em^r^, *cre* gene cloned at the *Eco*RI and *Hind*III sites (c*at* gene deletion vector)	[53]
pNZ8048	Cm^r^, pNZ8048 derivative containing the promoter P8	[61]
pNZ8048-*nisZ*	Cm^r^, pNZ8048 derivative containing the promoter P8/*nisZ*	[61]
pLEB124	Em^r^, *L. lactis* secretion vector harboring lactococcal promoter P45	[27]
pLEB672	Em^r^, pLEB124 derivative containing the *nisFEG* gene	[62]
pLEB124-*nisIFEG*	Em^r^, pLEB124 derivative containing the *nisIFEG* gene	From Per Saris’ lab
pLEB674	Em^r^, pLEB124 derivative containing the *nisRKFEG* gene	[62]

### DNA manipulations and cloning

Restriction enzymes, DNA markers, DNA ligase, DNA polymerases and DNA gel extraction kit were purchased from Takara Bio. Inc. (Dalian, China). Bacterial genome rapid extraction part A kit was purchased from Spark Jade (China). Polymerase chain reaction (PCR) product purification kit was purchased from Thermo Fisher Scientific (Waltham, USA). The commercial nisin was purchased from Sigma (St. Louis, USA). Chromosomal DNA plasmid DNA, and total RNA of *L. lactis* N8 or *E. coli* were isolated using QIAprep spin kit (small scale) following the manufacturer’s instructions. PCR was performed on Bio-Rad S1000 Thermal Cycler (Bio-Rad Laboratories, Inc. USA). Primers used in this study were purchased from GENEWIZ (Suzhou, China), and are listed in Table 3. Recombinant plasmids were introduced into *L. lactis* by electroporation using the Bio-Rad Gene Pulser (Bio-Rad Laboratories, Richmond, USA). Plasmids were introduced into *E. coli* through the CaCl_2_ method [52].

**Table 3:** Primers used in this study

Primers	Sequence (5′-3′)
For the construction of deletion vector (pNZ5319-up-down)	
Up-f	CCGCTCGAGTTAATCGGTGGTGTTACTACTGG
Up-r	CCCATTTAAATTTTAACCGGGGTTTTTGC
Down-f	CCCGAGCTCCTGAAGCGGGAGATACAGAAAC
Down-r	GAAGATCTCGCTTCAATCTCTCCCAAAGT
For the identification of mutant strain (*L. lactis* N8-1)	
Inner-f	AAATATGGAATTGAAGCATTTAA
Inner-r	TTATTCTTTCGGTTTAGATGACT
Exter-f	TGAATAAGAATTTGAACCCTTTA
Exter-r	AGAAATTCTCTGATAAATTTTCTG
Cat-f	TCAAATACAGCTTTTAGAACTGG
Cat-r	TACAGTCGGCATTATCTCATATTA
Ery-f	CTTGCTCATAAGTAACGGTAC
Ery-r	CGATACCGTTTACGAAATTGG
For the RT-qPCR	
Q-tufA-f	GACCTCTTGAGCGAATACGACT
Q-tufA-r	TTCTTCAACTTTAGCAACCCATT
Q-rpsN-f	CCTGCAAAATTCTCAACACAAGC
Q-rpsN-r	GCGAAGACAGATACGGCAAAGT
Q-rplR-f	TGGTACTAAAACTGAACAAGCCG
Q-rplR-r	TGCAACACGTCCGTGATAGAGGT
Q-csc2B-f	ACAGGAGGAGCACTTTCAATCG
Q-csc2B-r	GGTCCATCCATCCCAGGTT
Q-csc2C-f	GCCAGTTTACAGGCACAGGTC
Q-csc2C-r	GGATTAGCGTCATTCGTAGCATT
Q-pi339-f	TAGTCGCAGCAGGAATCTTTG
Q-pi339-r	TATCTTGATTGATTTGGTCTTTC
Q-pp423-f	CGCAATAAGAAGAATGCCAAGC
Q-pp423-r	GATGCCCCAGCGATCAATA
Q-butA-f	ATTATCAACGCAACCTCACAAGC
Q-butA-r	TCCCCATTCATCATCTTTACCAG
Q-butB-f	CCGTCAGCAGAACATCCTAATC
Q-butB-r	TCAGCCAATCCTCCACCAT
Q-arcA-f	AAGCGGGTTACCGTCCAG
Q-arcA-r	TTGGTAATGGGTTGAGGTAGAAA
Q-arcB-f	GCTCCAGATTCACTTCATCCTAC
Q-arcB-r	TCTTCCCAGTTAGATTCTCCCAT
Q-galM-f	AATTGTTCGTGGTGATATCGTTG
Q-galM-r	ATCACAATACTTGGTTGGTCGGT
Q-galK-f	TCCTATTGGACTGTAACACTCTA
Q-galK-r	TTCATCCCCAATCAAATCAGTAT
Q-lacZ-f	CGGTCCGCTGCTCTCATTATCCT
Q-lacZ-r	AGTCATTCCGTGCGTTTCG

### Deletion of PRF DNA region

The vector for the deletion of the PRF was constructed as described in our previous work [53]. Briefly, two fragments (upstream homology arm 1555 bp, downstream homology arm 1414 bp) of the flanking region of PRF were amplified by PCR with a proof-reading polymerase (Takara) and the *L. lactis* N8 chromosome was used as template. Then, the fragments were ligated into the *Xho*I–*Swa*I and *Sac*I–*Bgl*II restriction sites of pNZ5319. The recombinant plasmid pNZ5319-up-down obtained was transformed into *E. coli* DH5α cells strain by CaCl_2_ method [52]. After verifying the accuracy of cloning (Additional file 1: Fig. S5), pNZ5319-up-down was isolated and electroporated into *L. lactis* N8 competent cells to generate mutant *L. lactis* N8-1-*cat*. After the deletion of the PRF, *cat* gene in the mutant was retrieved by introducing the plasmid pNZTS-Cre into *L. lactis* N8-1-*cat* strain. The final mutant was named as *L. lactis* N8-1 and used for further analysis.

### Genome resequencing

Isolation of genomic DNA was carried out using SDS method. Total DNA obtained was subjected to quality control by agarose gel electrophoresis and quantified by Qubit (Thermo). The genome of *L. lactis* N8-1 was sequenced with MPS (massively parallel sequencing) Illumina technology (Illumina, San Diego, USA). The DNA library was constructed: a paired-end library with an insert size of 350 bp. The 350-bp library was sequenced using an Illumina PE150 strategy. Library construction and sequencing were performed at the Beijing Novogene Bioinformatics Technology Co., Ltd. Quality control of paired-end reads were performed using in-house program.

#### Data processing

The original figure data obtained by high-throughput sequencing platform of Illumina PE150 (Illumina) were transformed into raw sequenced reads by CASAVA base calling software (version 1.8.2; Illumina, Inc. USA), and stored in FASTQ format, containing sequencing information and the corresponding sequencing quality information of the reads. The sequenced data were filtered, and the sequence of adapter and low-quality data were removed, resulting in the clean data used for subsequent analysis.

#### Reads mapping

The reads comparison is the basis of the resequencing analysis. The variation information of the sample and the reference is obtained by aligning the sample reads with the designated reference sequence (*L. lactis* N8 genome). Mapping the reads to the reference sequence using BWA software (version 0.7.17; http://bio-bwa.sourceforge.net), counting the coverage of the reference sequence to the reads and make explanations of the alignment results using the SAMTOOLS software (version 1.11; http://www.htslib.org).

#### SV (structural variation) analysis

SV refers to the insertion, deletion, inversion and translocation of the large segments in the genome level. The insertion, deletion, inversion, intra-chromosomal translocation, and inter-chromosomal translocation between the reference and the sample are found by Integrative Genomics Viewer software (version 2.6.3; https://igv.org). The variation map of the whole genome was created by Circos (version 0.6; http://circos.ca/software) to show reads coverage and the distribution of insertion and deletion information.

### Phenotype microarray analysis of the lactococcal strains (BIOLOG)

Phenotype microarray system (Biolog, California, USA) was used to determine the metabolism of wild-type and mutant strains with GP2 MicroPlate™ [4]. Sample preparation and assays were conducted following the manufacturer’s instructions. Briefly, a sterilized cotton swab was used to collect lactococcal cells from the surface of the solid medium and the cells were suspended in the inoculation (0.40% sodium chloride, 0.03% Pluronic F-68, and 0.02% Gellan Gum) (Biolog). The cell density of different strains was equalized, and 150 μL of the samples were pipetted into GP2 plate with various substrates. The plates were sealed with a cover and incubated in the OmniLog^®^ instrument (Biolog) at 30 °C for 24 h. The data were recorded automatically by the machine every 30 min (15 s of shaking before recording), and analyzed using OL-OM 3.0 software (Biolog).

### Assessment of growth profiles

For growth profile experiments, *L. lactis* strains were cultured at 30 ℃ for 6 h in static (non-aerated) condition to log phase, harvested by centrifugation (5000×*g*, 3 min), and washed twice with PBS (phosphate buffered saline, pH 7.4). After washing, the cells were resuspended (adjusted to the same initial cell concentration) in GM17 medium as seed. For the determination of growth curves at different temperatures, we transferred 200 μL of each sample to a Bioscreen honeycomb plate (100 wells). The growth profiles were monitored by measuring OD600 for 18 h at different temperatures (30 ℃, 37 ℃, 39 ℃) by using the Bioscreen C™ system (Lab-systems, Helsinki, Finland) [54]. For the determination of growth curves at different nisin concentrations, standard nisin was added at various concentrations (4000, 5000, 6000, 6500, 7000, and 7500 IU/mL, respectively) to GM17 medium to obtain six groups. Then, the seeds were inoculated at a concentration of 1%, and 200 μL of each sample were transferred to a Bioscreen honeycomb plate (100 wells). The growth profiles were monitored by measuring OD600 for 48 h at 30 ℃ by using the Bioscreen C™ system. For the determination of growth curves at different lactic acid concentrations, lactic acid [80% (v/v)] was added at various concentrations [0.24% (v/v), 0.32% (v/v), and, 0.40% (v/v), respectively)] to GM17 medium to obtain three groups. Then, the seeds were inoculated at a concentration of 1% and 200 μL of each sample were transferred to a Bioscreen honeycomb plate (100 wells). The growth profiles were monitored by measuring OD600 for 18 h at 30 ℃ by using the Bioscreen C™ system. The OD600 was measured at 15-min intervals, and static culture was gently agitated for 10 s before each measurement.

### Assessment of nisin immunity

The nisin immunity of wild-type and mutant strains was determined by the 96-well plate gradient dilution method [25] with minor modifications. Briefly, the wild-type and mutant strains were grown for 6 h in antibiotic-free GM17 medium, and then washed twice with PBS. After washing, the cells were resuspended in GM17 medium and adjusted to the same cell concentration, and then diluted to a ratio of 1:1000 as seed solution. Nisin was added with a gradient concentration of 2000–8000 IU/mL at intervals of 500 IU/mL. Subsequently, 2 μL of seed solution were added into 198 μL of gradient nisin medium, and the mixtures were incubated at 30 ℃ for 20 h in 96-well plate. The cell concentration data were collected by microplate reader (Synergy 2, BioTek Instruments, Inc.) at a wavelength of 600 nm. The nisin immunity of the strains was determined as the minimum nisin concentration needed to ensure that the turbidity did not change to > 10% at 600 nm. The next lower nisin concentration was determined as the nisin immunity level of the tested strain.

### Assessment of lysozyme and lactic acid tolerances

To determine lysozyme and lactic acid tolerances, the lactococcal cells were cultured at 30 ℃ for 6 h in static (non-aerated) condition to log phase and harvested by centrifugation (5000×*g*, 3 min), washed twice with PBS, and resuspended in equal volume of GM17 medium. Lysozyme and lactic acid were added to the cell suspension at a final concentration of 10 mg/mL and 1.5% (v/v), respectively. Cell viability was determined at various time points (30, 60, 90, and 120 min) by colony counting. A total of 5 μL of serially diluted cell suspension were spotted on GM17 plates and cultured at 30 ℃ for 20 h, and then the plates were photographed. For survival rate experiments, colonies on plates containing 20–200 CFU were counted [44].

### Assessment of nisin yield

Nisin yield was determined by the agar well diffusion method [55] with minor modifications. Briefly, the broth of the tested strains after fermentation was boiled for 10 min and cells were removed by centrifugation at 8000 rpm for 3 min. Then, the supernatant was appropriately diluted with 0.02 M HCl. *M. luteus* (10^7^ CFU/mL) was used as indicator and inoculated at a concentration of 1% (v/v) into 30-mL melted/cooled LB agar. To enhance nisin diffusion, 1.5% (v/v) Tween 80 (JiangTian, Tianjin, China) was added to the medium and mixed well. Then, the medium was quickly poured into sterile plates. After solidification and pre-cultivation, a 7-mm-diameter sterile cork borer (MRS Scientific Ltd, Wickford, UK) was used to generate agar well for loading samples. Standard nisin solutions (concentrations of 20, 40, 80, 100, 200, and 400 IU/mL) were prepared using nisin powder. Subsequently, the standard nisin solutions and sample solutions were respectively loaded into the wells (80 μL per well), and the plates were incubated at 37 °C for 24 h. The diameter of inhibition zone was measured by calipers. A regression equation was derived from the nisin standard data.

### SEM

The lactococcal cells were cultured at 30 ℃ for 6 h to exponential phase, harvested at 8000 rpm for 2 min, and washed twice with PBS. The cells were resuspended in equal volume of GM17 medium (supplemented with lysozyme to a final concentration of 17.5 mg/mL) and incubated for 1 h and 2 h. The bacterial cells without lysozyme treatment were set as controls. After incubation, the cells were washed with PBS and fixed overnight in 2.5% glutaraldehyde (Sigma) at 4 ℃. Then, the cells were washed with PBS and dehydrated using gradient ethanol (50–100%, 15 min for each gradient). After dehydration, ethanol was replaced with tertiary butyl alcohol (Sigma). Subsequently, the samples were added onto the plate and prepared for SEM (QUANTA 200) observation [56].

### Flasks fermentation and fed-batch fermentation

After overnight cultivation, *L. lactis* N8 and *L. lactis* N8-1 were inoculated in GM17 medium for 6 h as seed cultures. To perform the flask fermentation experiments, 250-mL Erlenmeyer flasks containing 100 mL of GM17 fermentation medium were used. A total of 1 mL of the seed cultures was inoculated into static flasks and incubated for 16 h at 30 °C. Samples were collected every 2 h for the analysis of cell density, fermentation broth pH, and nisin production. Fed-batch fermentation experiments were conducted at 30 °C for 16 h. Initially, the pH of the three groups of fermentation medium was adjusted to pH 6.0, 5.5, and 5.0 with 80% (v/v) lactic acid, respectively. Then, three groups of media were inoculated with 1% of seed culture. The pH of the three fermentation broths was controlled at 6.0, 5.5, and 5.0 by adding 10 M NaOH, respectively. The fermentation broths were sampled every 2 h for the analysis of cell density, fermentation broth pH, and nisin production.

### TMT quantitative proteomics and analysis

TMT-labeled quantitative proteomics technology was applied to reveal changes in protein synthesis of *L. lactis* N8-1 and perform functional analysis of differentially synthetized proteins. The TMT quantitative proteomics of *L. lactis* N8 and *L. lactis* N8-1 were performed by Shanghai Majorbio Bio-pharm Technology Co., Ltd. (Shanghai, China) as following:

#### Total protein extraction

The *L. lactis* samples were suspended with protein lysis buffer (1% SDS, 200 mM DTT, 50 mM Tris–HCl, pH 8.8) which included appropriate protease inhibitor to inhibit protease activity, and treated for three times by high throughput tissue crusher Wonbio-96c (WanBo biotechnology co., Ltd, Shanghai, China) for 40 s. Then, the mixture was incubated on ice for 30 min during which the samples were vortexed 5–10 s every 5 min. Samples were incubated at 100 ℃ for 10 min, then transferred on ice for 30 min. After that, samples were centrifuged at 12,000×*g* for 20 min at 4 ℃, and supernatants were collected. Five volumes of pre-cooled acetone (Sigma) were added and proteins were precipitated at – 20 ℃ overnight. The second day, the solution was centrifuged at 12,000×*g* for 20 min at 4 ℃. The supernatant was discarded and the precipitation was washed twice with 90% pre-cooled acetone. Lysis buffer (1% SDS, 8 M urea, cocktail) was used to resuspend the precipitation, then samples were centrifugated at 12,000×*g* for 20 min at 4 °C. The protein concentration in the supernatant was determined by bicinchoninic acid (BCA) method using BCA Protein Assay Kit (Beyotime biotechnology, Shanghai, China).

#### Protein digestion and TMT labeling

One hundred microgram of proteins were resuspended with tetraethylammonium bromide (Haihang Industry, Jinan, China) at a final concentration of 100 mM. The mixture was reduced with tris (2-carboxyethyl) phosphine (Sigma) at a final concentration of 10 mM at 37 °C for 60 min, and alkylated with iodoacetamide (Sigma) at a final concentration of 40 mM at room temperature for 40 min in darkness. Six fold volumes of cold acetone were added to precipitate protein at − 20 °C for 4 h. After centrifugation at 10,000×*g* at 4 °C for 20 min, the pellet was resuspended with 100 μL 50 mM riethylammonium bicarbonate buffer (Sigma). Trypsin was added at 1:50 trypsin-to-protein mass ratio and incubated at 37 °C overnight. Trypsin-digested peptides were labeled with 10-plex TMT reagents (Thermo) according to the manufacturer’s instructions. Briefly, one unit of TMT reagent were thawed and reconstituted in 50 μL acetonitrile (Sigma). After tagging for 2 h at room temperature, hydroxylamine (Thermo) was added to react for 15 min at room temperature. Finally all samples were pooled, desalted and vacuum-dried.

#### High pH RPLC separation and LC–MS/MS analysis

The pooled samples were fractionated into fractions by ACQUITY Ultra Performance liquid chromatography (Waters, USA) with ACQUITY UPLC BEH C18 Column (1.7 μm, 2.1 mm × 150 mm, Waters, USA) to increase proteomic depth. Briefly, peptides were first separated with a gradient of elution (Phase B: 5 mM ammonium hydroxide solution containing 80% acetonitrile, pH 10) over 66 min at a flowrate of 200 μL/min. Twenty fractions were collected from each sample, which were subsequently pooled, resulting in ten total fractions per sample. Then, trypsin-digested peptides were analyzed by online nano flow liquid chromatography tandem mass spectrometry performed on an EASY-nLC system (Thermo Fisher Scientific) connected to a Q Exactive quadrupole orbitrap mass spectrometer (Thermo) through a nanoelectrospray ion source. Briefly, the C18-reversed phase column (75 μm × 25 cm, Thermo Fisher Scientific) was equilibrated with solvent A (A: 2% ACN with 0.1% formic acid) and solvent B (B: 80% ACN with 0.1% formic acid). The peptides were eluted using the following gradient: 0–1 min, 0–5% B; 1–63 min, 5–23% B; 63–88 min, 23–48% B; 88–89 min, 48–100% B; and 89–95 min, 100% B. The tryptic peptides were separated at a flow rate of 300 nL/min. The Q Exactive plus instrument was operated in the data-dependent acquisition mode (DDA) to automatically switch between full scan MS and MS/MS acquisition. The survey of full scan MS spectra (m/z 350–1300) was acquired in the Orbitrap with 70,000 resolution. The automatic gain control (AGC) target at 1e6 and the maximum fill time was 50 ms. Then the top 20 most intense precursor ions were selected into collision cell for fragmentation by higher-energy collision dissociation (HCD). The MS/MS resolution was set at 35,000 (m/z 100), the automatic gain control (AGC) target at 1e5, the maximum fill time at 100 ms, and dynamic exclusion was 18 s.

#### Protein identification

MS/MS spectra were searched using Protein Discoverer™ Software 2.1 (Thermo Fisher Scientific) against *Streptococcus lactis* database and the decoy database as the following parameters. The highest score for a given peptide mass (best match to that predicted in the database) was used to identify parent proteins. The parameters for protein searching were set as follows: tryptic digestion with up to two missed cleavages, carbamidomethylation of cysteines and the TMT of N-terminus and lysine side chains of peptides as a fixed modification, and oxidation of methionines and protein N-terminal acetylation as variable modifications. Peptide spectral matches were validated based on Q-values at a 1% false discovery rate (FDR). Only the proteins which has at least one unique peptide was used for protein identifications. Proteins that changed more than 1.5-fold (up or down regulated) with a *P*-value less than 0.05 between *L. lactis* N8-1 and *L. lactis* N8 were deemed significantly difference proteins. The Uniprot database (https://www.uniprot.org/) and Pfam (http://pfam.xfam.org) were used for protein sequence search, alignment analysis, and protein domain prediction. The databases used for metabolic pathway search and analysis were iPath3.0 (https://pathways.embl.de/) and KEGG (https://www.genome.jp/kegg/). NCBI BLASTp (https://blast.ncbi.nlm.nih.gov/Blast.cgi?PROGRAM=blastp&PAGETYPE=BlastSearch&LINK_LOC=blasthome) was used for protein function domain prediction. Subcellular location database (https://abi-services.informatik.uni-tuebingen.de/yloc/webloc.cgi) was used to predict the location where proteins appear in the cell.

### Quantitative real-time PCR

*Lactococcus lactis* N8 and *L. lactis* N8-1 were cultured in GM17 medium for 6 h and appropriately diluted to achieve similar cell density. Then, the total RNA was extracted and reversed-transcribed into first-strand cDNA using RevertAid First Strand cDNA Synthesis Kit (Thermo Fisher Scientific). The gene transcription levels of *rpsN, rplR, csc2B, csc2C, pi339, pp423, butA, butB, arcA, arcB, galM, galK and lacZ* were then assessed through RT-qPCR to confirm the proteomics data. The *tufA* gene was selected as housekeeping gene [57] and comparative CT(2^**−**ΔΔ*C*T^) method was employed for data analysis. Transcription with more than two-fold change was regarded as statistically significantly different.

### Statistical analysis

The experiments to determine the growth profiles of *L. lactis* under different treatments (nisin, temperature, and lactic acid) were performed in independent biological triplicates, and each sample was additionally collected in technical triplicates. The experiments to determine the survival rates of *L. lactis* under different treatments (lysozyme and lactic acid) were independently repeated at least three times. The data are shown as mean ± standard deviation (SD). Assays to determine the nisin immunity of *L. lactis* in 96-well plate and nisin yield of different *L. lactis* strains under diverse treatments (pH 6.0, 5.5, 5.0) were independently repeated at least three times. The difference between two groups was compared by the Student’s *t*-test and values with *P* < 0.05 were considered significant. RT-qPCR experiments were independently repeated at least three times, and the data are given as mean ± SD. Statistical analyses of the data were performed using Origin 85 software version 8.5.0 SRI (OriginLab Corporation, USA) and GraphPad Prism 5 software version 5.01 (GraphPad software, Inc.).

## Supplementary Information

**Additional file 1: Fig. S1.** KEGG functional enrichment (*L. lactis* N8-1 vs *L. lactis* N8). a Upregulated proteins (> 1.2-fold change with *P* < 0.05); b downregulated proteins (> 1.2-fold change with *P* < 0.05). **Fig. S2.** Gene Ontology (GO) function annotations. a all proteins; b upregulated proteins (> 1.2-fold change with *P* < 0.05); c downregulated proteins (> 1.2-fold change with *P* < 0.05). **Fig. S3.** Diagram of protein subcellular localization. Red bars indicate upregulated proteins (> 1.2-fold change with *P* < 0.05), green bars indicate downregulated proteins (> 1.2-fold change with *P* < 0.05). **Fig. S4.** RT-qPCR analysis of genes with higher and lower expression level. *rpsN, rplR*: ribosomal protein; *csc2B, csc2C*: cell surface protein; *pi339, pp423*: bacteriophage protein; *butA, butB*: butanoate metabolism protein; *arcA, arcB*: arginine biosynthesis protein; *galM, galK*: galactose metabolism protein: *lacZ*; beta-galactosidase. **Fig. S5.** PCR verification of the knockout vector construction. **Table S1.** Gene content of deleted PRF. **Table S2.** Substrate consumption ratio. **Table S3.** Nisin-immunity of engineered strains. **Table S4.** Upregulated proteins (> 1.2-fold change with *P* < 0.05). **Table S5.** Downregulated proteins (> 1.2-fold change with *P* < 0.05).

## Data Availability

All data generated or analyzed during this study are included in this published article and its additional file.

## Abbreviations

LAB: Lactic acid bacteria
gTME: Global transcription machinery engineering
PRF: Prophage-related fragment
MS: Mass spectrometry
2-DE: Two-dimensional gel electrophoresis
TMT: Tandem mass tags
HSP: Heat shock protein
CSP: Cold shock protein
BCA: Bicinchoninic acid
SD: Standard deviation
