# Supplementary material for: Engineering Lactococcus lactis as a multi-stress tolerant biosynthetic chassis by deleting the prophage-related fragment
Source: Microb Cell Fact. 2020 Dec 9;19:225. doi: 10.1186/s12934-020-01487-x (PMC7727215; doi:10.1186/s12934-020-01487-x)
Supplement: Supplementary file 1 — Additional file 1: Fig. S1. KEGG functional enrichment (L. lactis N8-1 vs L. lactis N8). a Upregulated proteins (> 1.2-fold change with P < 0.05); b downregulated proteins (> 1.2-fold change with P < 0.05). Fig. S2. Gene Ontology (GO) function annotations. a all proteins; b upregulated proteins (> 1.2-fold change with P < 0.05); c downregulated proteins (> 1.2-fold change with P < 0.05). Fig. S3. Diagram of protein subcellular localization. Red bars indicate upregulated proteins (> 1.2-fold change with P < 0.05), green bars indicate downregulated proteins (> 1.2-fold change with P < 0.05). Fig. S4. RT-qPCR analysis of genes with higher and lower expression level. rpsN, rplR: ribosomal protein; csc2B, csc2C: cell surface protein; pi339, pp423: bacteriophage protein; butA, butB: butanoate metabolism protein; arcA, arcB: arginine biosynthesis protein; galM, galK: galactose metabolism protein: lacZ; beta-galactosidase. Fig. S5. PCR verification of the knockout vector construction. Table S1. Gene content of deleted PRF. Table S2. Substrate consumption ratio. Table S3. Nisin-immunity of engineered strains. Table S4. Upregulated proteins (> 1.2-fold change with P < 0.05). Table S5. Downregulated proteins (> 1.2-fold change with P < 0.05). [file 12934_2020_1487_MOESM1_ESM.doc]

**Additional file**

**Engineering *Lactococcus lactis* as a multi-stress tolerant** **biosynthetic chassis by deleting the** **prophage-related fragment**

Wanjin Qiao^1,2^, Yu Qiao^1^, Fulu Liu^1^, Yating Zhang^1^, Ran Li^2^, Zhenzhou Wu^3^, Haijin Xu^1*^, Per Erik Joakim Saris^2^ and Mingqiang Qiao^1*^

^1^Key Laboratory of Molecular Microbiology and Technology, Ministry of Education, Nankai University, Tianjin, China,

^2^Department of Microbiology, Faculty of Agriculture and Forestry, University of Helsinki, Helsinki, Finland

^3^State Key Laboratory of Medicinal Chemical Biology & Tianjin Key Laboratory of Protein Sciences, College of Life Sciences, Nankai University, Tianjin, China

*** Correspondence:**
Mingqiang Qiao, the Key Laboratory of Molecular Microbiology and Technology, Ministry of Education, College of Life Sciences, Nankai University, No.94 Weijin Road, Nankai District, Tianjin, 300071, China

[qiaomq@nankai.edu.cn](mailto:qiaomq@nankai.edu.cn) Tel: +862223503692 Fax: +862223503692

Haijin Xu, the Key Laboratory of Molecular Microbiology and Technology, Ministry of Education, College of Life Sciences, Nankai University, No.94 Weijin Road, Nankai District, Tianjin, 300071, China

nkxuhaijin@163.com

**
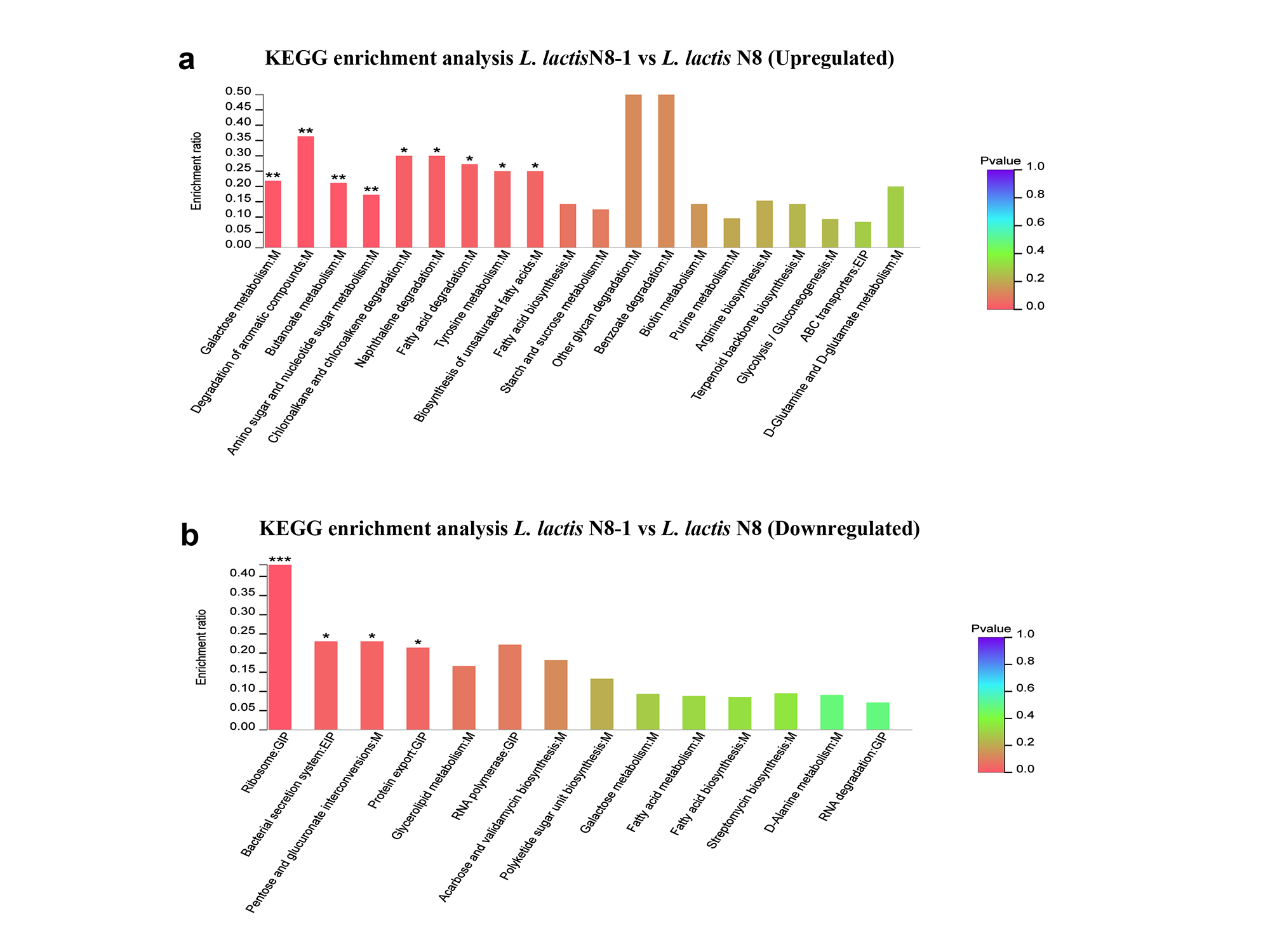
**

**Fig. S1** KEGG functional enrichment (*L. lactis* N8-1 vs *L. lactis* N8) **a** upregulated proteins (>1.2-fold change with *P<*0.05); **b** downregulated proteins (>1.2-fold change with *P<*0.05).

**
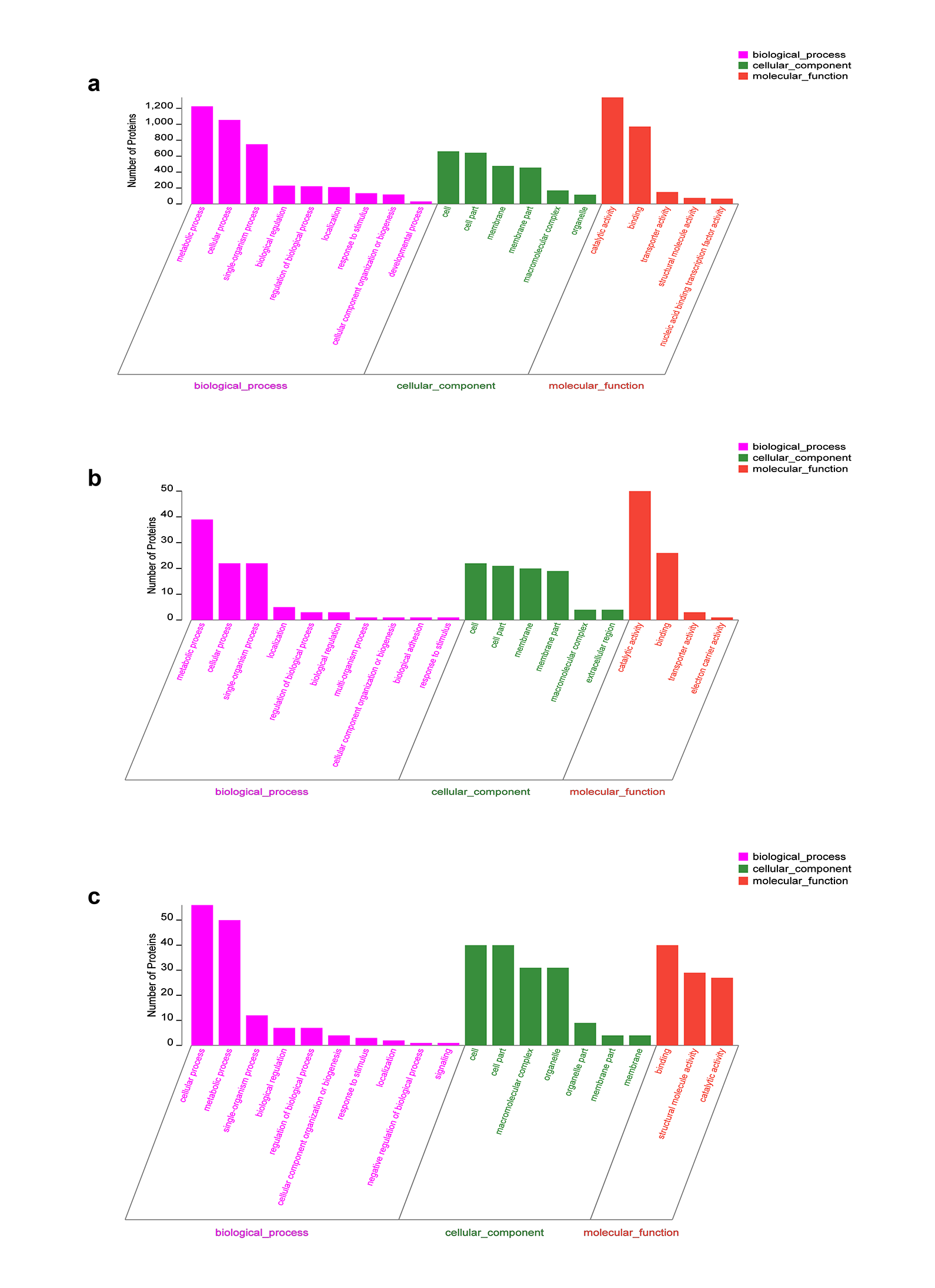
**

**Fig. S2** Gene Ontology (GO) function annotations for **(A)** all proteins; **(B)** upregulated proteins (>1.2-fold change with *P<*0.05); **(C)** downregulated proteins (>1.2-fold change with *P<*0.05).

**
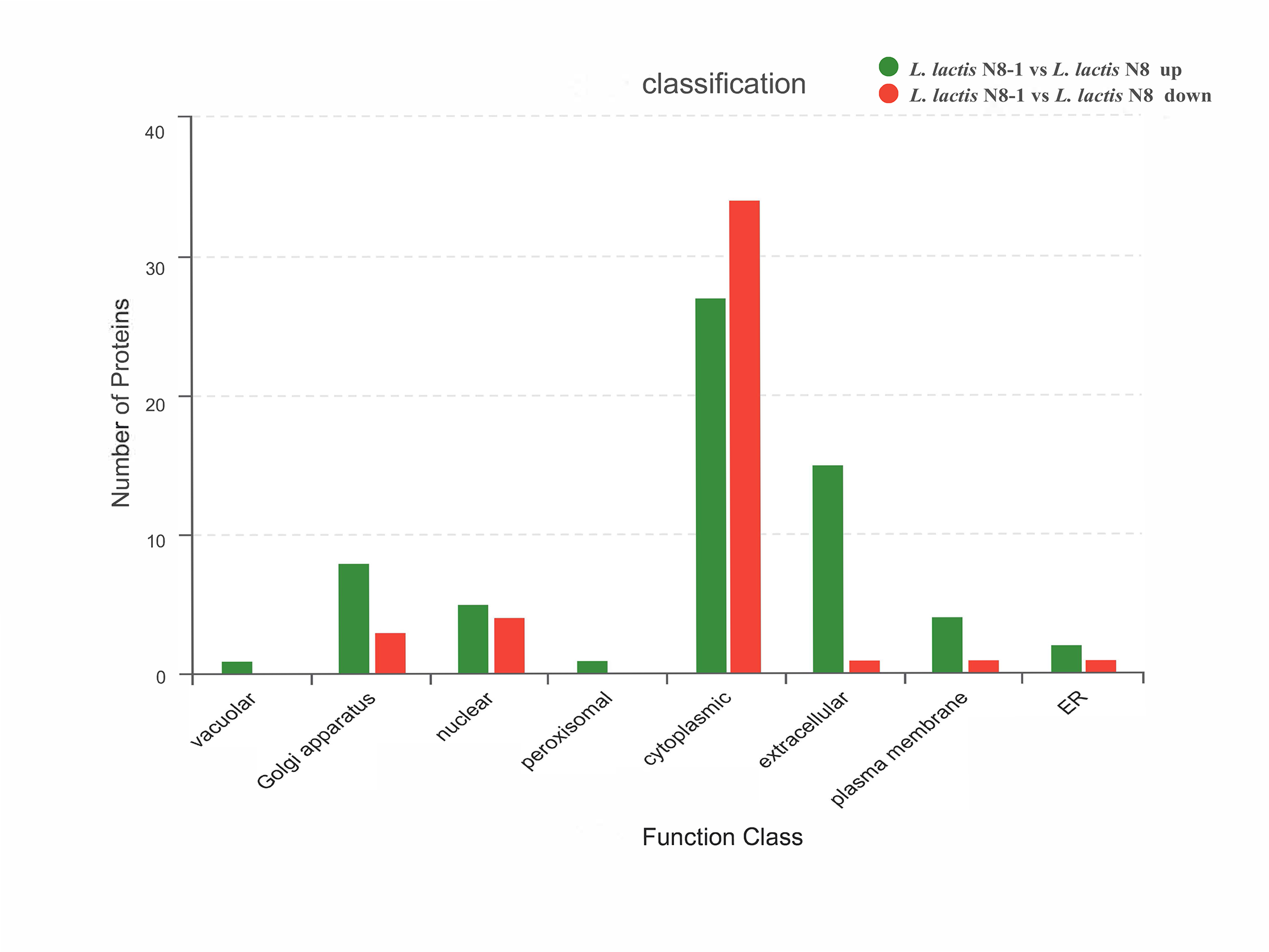
**

**Fig. S3** Diagram of protein subcellular localization. Red bars indicate upregulated proteins (>1.2-fold change with *P<*0.05), green bars indicate downregulated proteins (>1.2-fold change with *P<*0.05).


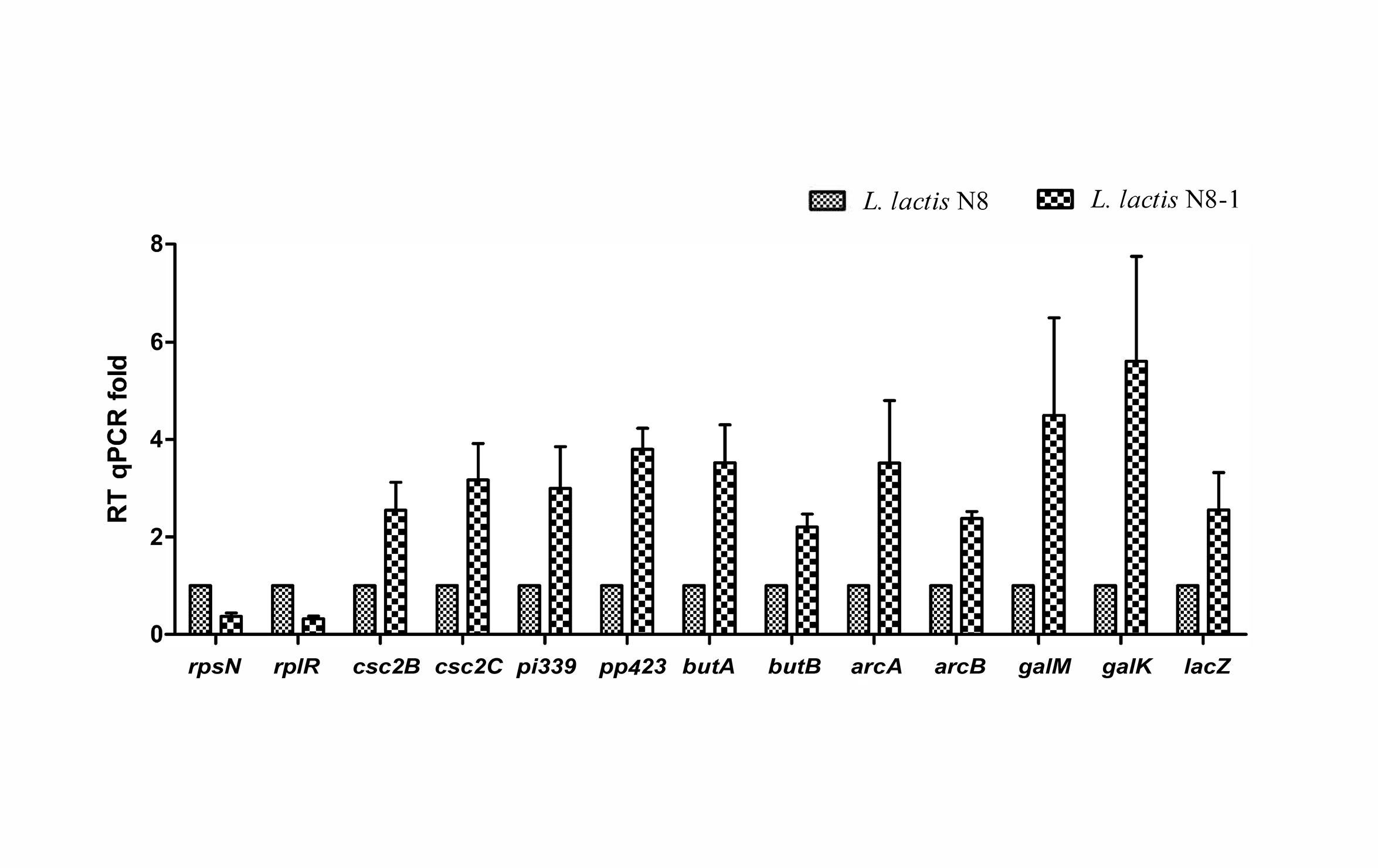


**Fig. S4** RT-qPCR analysis of genes with higher and lower expression level. *rpsN, rplR*: ribosomal protein; *csc2B, csc2C*: cell surface protein; *pi339, pp423*: bacteriophage protein; *butA, butB*: butanoate metabolism protein; *arcA, arcB*: arginine biosynthesis protein; *galM, galK*: galactose metabolism protein: *lacZ*; beta-galactosidase.

**
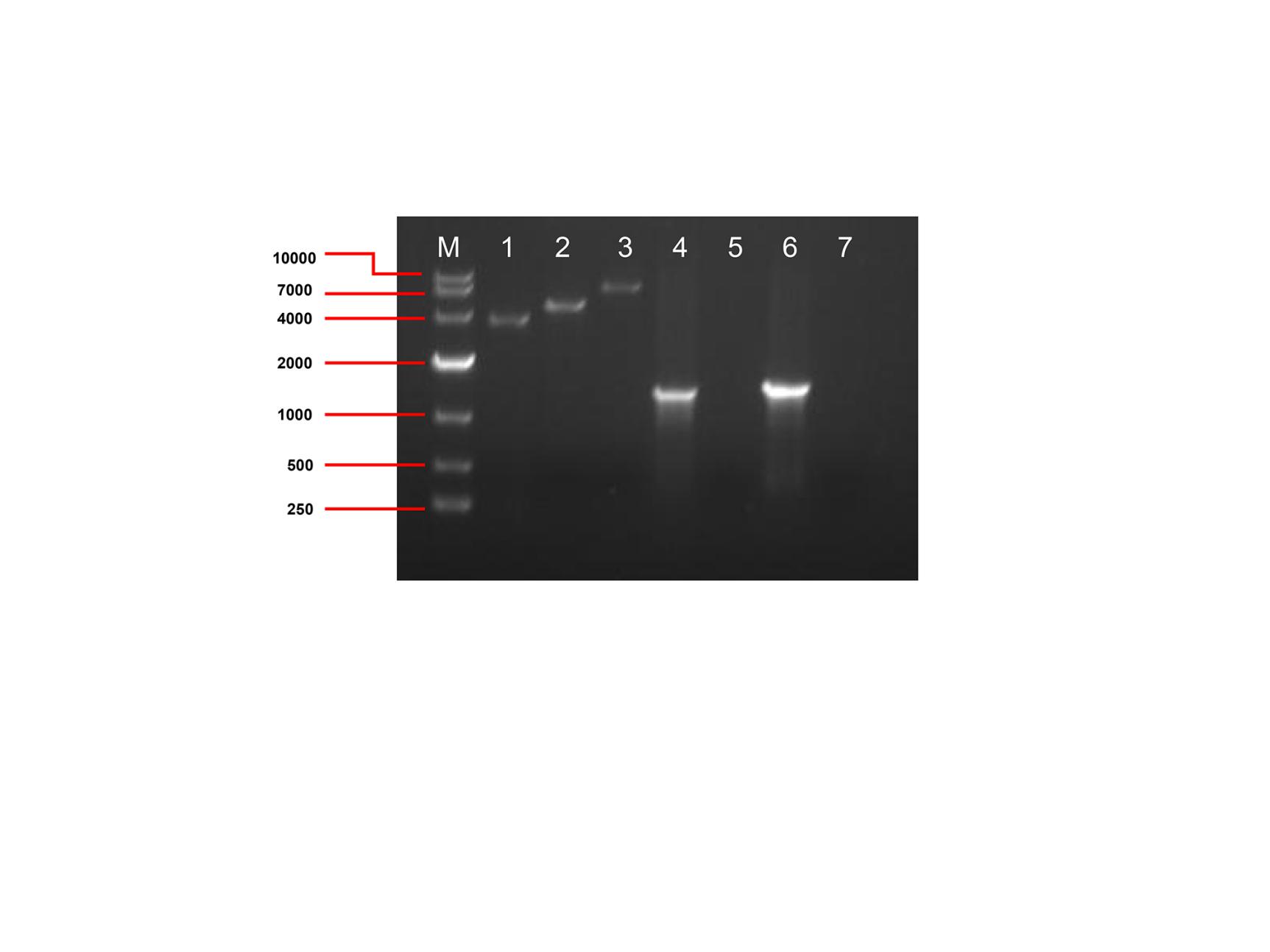
**

**Fig. S5** PCR verification of the knockout vector construction.

M: DL10000 DNA Marker;

Lane 1: pNZ5319 digested by restriction endonuclease *Xho*I (3,737 bp);

Lane 2: pNZ5319-down digested by restriction endonuclease *Xho*I (5,151 bp);

Lane 3: pNZ5319-up-down digested by restriction endonuclease *Xho*I (6,706 bp);

Lane 4: pNZ5319-up-down used as template and the primers were Down-f/Down-r (1,414 bp);

Lane 5: pNZ5319 used as template and the primers were Down-f/Down-r;

Lane 6: pNZ5319-up-down used as template and the primers were Up-f/Up-r (1,555 bp);

Lane 7: pNZ5319 used as template and the primers were Up-f/Up-r.

**Table S1. Gene content of deleted PRF**

| **Gene order** | **Description** | **Probability** | **E-value** | **Hit** |
| --- | --- | --- | --- | --- |
| 1 | BRK domain | 86.79 | 0.19 | [PF07533.17](http://pfam.xfam.org/family/PF07533.17#tabview=tab0) |
| 2 | Flagellar and Swarming motility protein | 59.88 | 4 | [PF06289.12](http://pfam.xfam.org/family/PF06289.12#tabview=tab0) |
| 3 | Bacterial protein of unknown function (DUF961) | 99.83 | 2.9e-20 | [PF06125.12](http://pfam.xfam.org/family/PF06125.12#tabview=tab0) |
| 4 | FtsK/SpoIIIE family protein | 99.53 | 4e-13 | [PF01580.19](http://pfam.xfam.org/family/PF01580.19#tabview=tab0) |
| 5 | Replication initiation factor | 100 | 2.5e-32 | [PF02486.20](http://pfam.xfam.org/family/PF02486.20#tabview=tab0) |
| 6 | Domain of unknown function | 65.9 | 4.9 | [PF15930.6](http://pfam.xfam.org/family/PF15930.6#tabview=tab0) |
| 7 | Domain of unknown function (DUF4363) | 87.19 | 2 | [PF14276.7](http://pfam.xfam.org/family/PF14276.7#tabview=tab0) |
| 8 | Putative phage protein | 65.81 | 1.1 | [PF13009.7](http://pfam.xfam.org/family/PF13009.7#tabview=tab0) |
| 9 | Antirepressor | 99.28 | 1.3e-11 | [PF14156.7](http://pfam.xfam.org/family/PF14156.7#tabview=tab0) |
| 10 | BetR domain | 98.3 | 0.0000022 | [PF08667.11](http://pfam.xfam.org/family/PF08667.11#tabview=tab0) |
| 11 | Thioester domain | 98.01 | 0.0000056 | [PF08341.12](http://pfam.xfam.org/family/PF08341.12#tabview=tab0) |
| 12 | Uncharacterized protein conserved in bacteria | 91.32 | 0.45 | [PF09954.10](http://pfam.xfam.org/family/PF09954.10#tabview=tab0) |
| 13 | Conjugative transposon protein | 99.96 | 2.9e-28 | [PF12642.8](http://pfam.xfam.org/family/PF12642.8#tabview=tab0) |
| 14 | Domain of unknown function (DUF5665) | 82.98 | 3 | [PF18910.1](http://pfam.xfam.org/family/PF18910.1#tabview=tab0) |
| 15 | TcpE family protein | 99.73 | 2.8e-17 | [PF12648.8](http://pfam.xfam.org/family/PF12648.8#tabview=tab0) |
| 16 | AAA-like domain | 99.98 | 6.8e-30 | [PF12846.8](http://pfam.xfam.org/family/PF12846.8#tabview=tab0) |
| 17 | TrbL/VirB6 plasmid conjugal transfer protein | 98.06 | 0.0013 | [PF04610.15](http://pfam.xfam.org/family/PF04610.15#tabview=tab0) |
| 18 | CHAP domain | 97.78 | 0.00018 | [PF05257.17](http://pfam.xfam.org/family/PF05257.17#tabview=tab0) |
| 19 | Dynamitin | 76.18 | 14 | [PF04912.15](http://pfam.xfam.org/family/PF04912.15#tabview=tab0) |
| 20 | Antirestriction protein | 99.88 | 3.4e-22 | [PF07275.12](http://pfam.xfam.org/family/PF07275.12#tabview=tab0) |
| 21 | Putative excisionase (DUF1233) | 99.16 | 1.9e-10 | [PF06806.13](http://pfam.xfam.org/family/PF06806.13#tabview=tab0) |
| 22 | Phage integrase | 99.87 | 2.4e-21 | [PF00589.23](http://pfam.xfam.org/family/PF00589.23#tabview=tab0) |

**Table S2. Substrate consumption ratio**

| **Substrate** | ***L.lactis* N8** | ***L.lactis* N8-1** |
| --- | --- | --- |
| dextnin | 193 | 148 |
| tween80 | 30 | 32 |
| N-acelyl-D-glucosamine | 721 | 808 |
| cellbiose | 164 | 135 |
| D-fructose | 903 | 762 |
| α-D-glucose | 863 | 882 |
| maltotriose | 161 | 136 |
| D-mannose | 771 | 893 |
| sucrose | 803 | 869 |
| D-trehalose | 167 | 92 |
| methyl pyruvate | 71 | 56 |
| adenosine | 263 | 222 |

**Table S3. Nisin-immunity of engineered strains**

| **Strains** | **Nisin immunity(IU/mL)** |
| --- | --- |
| *L. lactis* N8 | 4500±204.1 |
| *L. lactis* N8-pNZ8048 | 4333±107.6 |
| *L. lactis* N8-pNZ8048-*nisZ* | 4250±204.1 |
| *L. lactis* N8-pLEB124 | 4416±107.6 |
| *L. lactis* N8-pLEB672 | 4666±107.6 |
| *L. lactis* N8-pLEB124-*nisIFEG* | 4833±107.6 |
| *L. lactis* N8-pLEB674 | 4583±107.6 |
| *L. lactis* N8-1 | 6500±204.1 |
| *L. lactis* N8-1-pNZ8048 | 6416±107.6 |
| *L. lactis* N8-1-pNZ8048-*nisZ* | 6333±107.6 |
| *L. lactis* N8-1-pLEB124 | 6416±107.6 |
| *L. lactis* N8-1-pLEB672 | 6666±107.6 |
| *L. lactis* N8-1-pLEB124-*nisIFEG* | 6750±204.1 |
| *L. lactis* N8-1-pLEB674 | 6500±204.1 |

**Table S4. Upregulated proteins (>1.2-fold change with P<0.05).**

| **Protein name** | **Description** | **fc(N8-1/N8)** | **P-value(N8-1/N8)** |
| --- | --- | --- | --- |
| YbeF | Collagen binding domain-containing protein | 2.374525 | 0.00268 |
| Csc2B | Cell surface protein | 2.148837 | 0.004267 |
| HMPREF0877_0863 | Uncharacterized protein | 2.127576 | 0.002516 |
| ------ | Uncharacterized protein | 2.071691 | 0.007244 |
| Csc2B1 | WxL domain-containing protein | 2.068097 | 0.005522 |
| RibG | YhgE/Pip domain-containing protein | 2.03235 | 0.02129 |
| YqcD | WxL domain-containing protein | 2.026217 | 0.004783 |
| YmjF | UDP-N-acetylglucosamine 2-epimerase | 2 | 0.01237 |
| GalK | Galactokinase | 1.982891 | 0.02836 |
| Pip | Phage infection protein | 1.982659 | 0.02092 |
| HMPREF8578_0259 | Phage tail protein | 1.942551 | 0.001115 |
| Pp423 | Phage transcriptional regulator, ArpU family | 1.941292 | 0.001169 |
| Csc2C | Uncharacterized protein | 1.867837 | 0.01248 |
| GalM | Aldose 1-epimerase | 1.831904 | 0.03289 |
| Pi308 | Prophage pi3 protein 34 | 1.775926 | 0.009255 |
| Pi339 | Prophage pi3 protein 39 | 1.760267 | 0.008765 |
| LacZ | Beta-galactosidase | 1.759325 | 0.03768 |
| TnpA2B2-ISLL6 | Transposase | 1.739785 | 0.01036 |
| YqbH | Putative transcriptional regulator | 1.732743 | 0.0358 |
| GalT | Galactose-1-phosphate uridylyltransferase | 1.677477 | 0.02862 |
| Phi3396 | Phage major capsid protein | 1.656615 | 0.002207 |
| ------ | Prophage protein | 1.653465 | 0.007825 |
| LacA | Galactoside O-acetyltransferase | 1.646409 | 0.03072 |
| ChiA | Chitinase | 1.629126 | 0.002192 |
| YmjE | Glycosyl transferase | 1.595777 | 0.0187 |
| LacS | Lactose and galactose permease GPH translocator family | 1.591603 | 0.006039 |
| AntB | Phage antirepressor Ant | 1.587258 | 0.01764 |
| YuaE | Aspartate protease | 1.571354 | 0.005723 |
| Pp261 | Uncharacterized protein | 1.554502 | 0.009853 |
| Csc2A | Cell surface protein | 1.552502 | 0.00115 |
| ------ | Uncharacterized protein | 1.551 | 0.000896 |
| YhjA | Uncharacterized protein | 1.544643 | 0.01194 |
| LLKF_0934 | Uncharacterized protein | 1.526839 | 0.000136 |
| YjdI | YjdI-like protein | 1.491592 | 0.01069 |
| ButB | 2,3-butanediol dehydrogenase S-alcohol forming (R)-acetoin-specific / Acetoin (Diacetyl) reductase | 1.491159 | 0.009199 |
| DltE | Short-chain dehydrogenase (D-alanine transfer protein) | 1.4842 | 0.01973 |
| ------ | Cytochrome c-type biogeneis protein CcmI | 1.473988 | 0.009346 |
| ClpP | ATP-dependent Clp protease proteolytic subunit | 1.472208 | 0.04425 |
| CVCAS_0073 | Uncharacterized protein | 1.469613 | 0.00055 |
| RpoB | DNA-directed RNA polymerase subunit beta | 1.468432 | 0.01676 |
| NifJ | Ferredoxin | 1.461262 | 0.008014 |
| ------ | Uncharacterized protein | 1.452792 | 0.004246 |
| YpcCD | Endo-beta-N-acetylglucosaminidase | 1.443026 | 0.02857 |
| XseB | Exodeoxyribonuclease 7 small subunit | 1.438259 | 0.007973 |
| RecT | Recombinase RecT | 1.435897 | 0.000246 |
| YieF | Copper chaperone | 1.433468 | 0.007102 |
| YneC | Uncharacterized protein | 1.425842 | 0.006549 |
| YwfB | TRAP-type C4-dicarboxylate transport system, small permease component | 1.425093 | 0.01906 |
| AcpP | Acyl carrier protein | 1.424756 | 0.008581 |
| YxbF | Kinesin-like protein | 1.424575 | 0.005104 |
| YiaA | Uncharacterized protein | 1.420889 | 0.01807 |
| HemH | Ferrochelatase | 1.420275 | 0.02511 |
| Pip | Phage infection protein | 1.404537 | 0.02076 |
| LACR_1693 | UDP-N-acetylglucosamine 1-carboxyvinyltransferase | 1.403581 | 0.01075 |
| YtbC | Uncharacterized protein | 1.403162 | 0.00831 |
| AcpP | Acyl carrier protein | 1.400409 | 0.04549 |
| CwlO | Secreted antigen GbpB/SagA/PcsB putative peptidoglycan hydrolase | 1.399364 | 0.02399 |
| YwiE | Uncharacterized protein | 1.394531 | 0.003565 |
| GreA | Transcription elongation factor GreA | 1.387922 | 0.01757 |
| LLNZ_06485 | Small integral membrane protein | 1.385507 | 0.007901 |
| YcjF | Uncharacterized protein | 1.382139 | 0.01635 |
| NisP | Epidermin leader peptide processing serine protease EPIP | 1.381128 | 0.04155 |
| NrdH | Glutaredoxin | 1.365879 | 0.005711 |
| YlcA | UPF0291 protein LK337_1552 | 1.36322 | 0.006945 |
| AmpC | Class A beta-lactamase-related serine hydrolase | 1.359343 | 0.006443 |
| ButA | 2,3-butanediol dehydrogenase, S-alcohol forming,(R)-acetoin-specific / Acetoin (Diacetyl) reductase | 1.354776 | 0.02273 |
| YjdB | Acyl-coenzyme A synthetases/AMP-(Fatty) acid ligases | 1.341274 | 0.02836 |
| YcfB | Transport permease protein | 1.33998 | 0.005649 |
| YedF | Beta-glucoside-specific PTS system IIABC component | 1.338834 | 0.008757 |
| LLKF_1366 | CO dehydrogenase maturation factor | 1.338078 | 0.002717 |
| YjdB | Uncharacterized protein | 1.324422 | 0.007491 |
| LLKF_0299 | Uncharacterized protein | 1.323308 | 0.01944 |
| LLKF_1856 | Cell envelope-associated transcriptional attenuator LytR-CpsA-Psr subfamily F2 | 1.321285 | 0.0237 |
| ------ | Uncharacterized protein | 1.319 | 0.000357 |
| AcmD | N-acetylmuramoyl-L-alanine amidase AmiB | 1.316588 | 0.02179 |
| AcmA | N-acetylmuramidase | 1.310488 | 0.04474 |
| YreE | Protease subunit of ATP-dependent Clp proteases | 1.307653 | 0.04955 |
| LLKF_1677 | Uncharacterized protein | 1.304259 | 0.03005 |
| YmgG | Gls24 family general stress protein | 1.303846 | 0.02494 |
| LeuB, IMDH;IDH1, IDH2, icd | Isocitrate dehydrogenase (NADP) | 1.294593 | 0.006425 |
| PflD | Formate acetyltransferase | 1.285642 | 0.01511 |
| YpcG | Sugar ABC transporter substrate-binding protein | 1.285573 | 0.02366 |
| YsiE | Glucan-binding repeat protein | 1.283351 | 0.02337 |
| ArcB | Ornithine carbamoyltransferase | 1.277524 | 0.01272 |
| ------ | Pyruvate formate-lyase | 1.275897 | 0.02517 |
| COLAER_02301 | Uncharacterized protein | 1.258211 | 0.005564 |
| MalF | Sugar ABC transporter permease | 1.256344 | 0.04914 |
| ------ | Uncharacterized protein | 1.254254 | 0.03259 |
| CVCAS_1938 | Uncharacterized protein | 1.250125 | 0.000245 |
| LL275_1908 | Uncharacterized protein | 1.245357 | 0.01667 |
| YhfB | Cyclic-di-AMP phosphodiesterase | 1.226834 | 0.03721 |
| Pfl | Pyruvate formate-lyase | 1.225974 | 0.04262 |
| YdbC | Seryl-tRNA synthetase | 1.224822 | 0.01919 |
| ArcA | Arginine deiminase | 1.221053 | 0.04788 |
| Pbg | Beta-glucosidase/6-phospho-beta-glucosidase/ beta-galactosidase | 1.220871 | 0.0432 |
| YbeA | IreB family regulatory phosphoprotein | 1.218718 | 0.009763 |
| YtaB | Gliding motility-associated protein GldE | 1.218623 | 0.01146 |
| YciB | D-alanyl-D-alanine carboxypeptidase | 1.212121 | 0.02305 |
| CspA | Cold-shock protein | 1.209953 | 0.03373 |
| TrePP | Maltose phosphorylase | 1.206864 | 0.04756 |
| LLKF_p0027 | Oleate hydratase | 1.202601 | 0.02796 |
| ------ | Bacteriocin | 1.202051 | 0.038 |
| YudB | Uncharacterized protein | 1.200405 | 0.003653 |

**Table S5. Downregulated proteins (>1.2- fold with *P<*0.05).**

| **Protein name** | **Description** | **fc(N8-1/N8)** | ***P*-value(N8-1/N8)** |
| --- | --- | --- | --- |
| ------ | Uncharacterized protein | 0.18932 | 0.00138 |
| ------ | Uncharacterized protein | 0.192722 | 0.000992 |
| ------ | Cell wall surface anchor family protein | 0.26405 | 0.001781 |
| ------ | Uncharacterized protein | 0.328542 | 0.003146 |
| ------ | Cell surface protein | 0.3595 | 0.000103 |
| ------ | Antirestriction protein ArdA | 0.388067 | 0.002062 |
| ------ | Putative transcriptional regulator | 0.465746 | 0.001791 |
| ------ | Hypothetical cell surface protein | 0.487154 | 0.01871 |
| RsmB | Transcription antitermination protein NusB | 0.663534 | 0.0478 |
| RpsU | 30S ribosomal protein S21 | 0.668468 | 0.005991 |
| RpmH | 50S ribosomal protein L34 | 0.675026 | 0.03698 |
| ------ | Uncharacterized protein | 0.677483 | 0.002906 |
| RpmI | 50S ribosomal protein L35 | 0.684836 | 0.01404 |
| ------ | Transcription regulator | 0.693684 | 0.02936 |
| ------ | Beta-1,6-galactofuranosyltransferase | 0.701113 | 0.03801 |
| RpsR | 30S ribosomal protein S18 | 0.705882 | 0.000186 |
| RplR | 50S ribosomal protein L18 | 0.707353 | 0.004683 |
| RpsP | 30S ribosomal protein S16 | 0.711156 | 0.009109 |
| VicR | Sensor histidine kinase | 0.711905 | 0.02939 |
| RpmG | 50S ribosomal protein L33 | 0.714358 | 0.004496 |
| RpsN | 30S ribosomal protein S14 type Z | 0.720812 | 0.009245 |
| RpsT | 30S ribosomal protein S20 | 0.722529 | 0.002713 |
| ------ | Uncharacterized protein | 0.725313 | 0.002501 |
| RpsP | 30S ribosomal protein S16 | 0.729783 | 0.002931 |
| ------ | Uncharacterized protein | 0.730892 | 0.04953 |
| RpmF | 50S ribosomal protein L32 | 0.743042 | 0.001378 |
| RpsI | 30S ribosomal protein S9 | 0.743056 | 0.0102 |
| RplU | 50S ribosomal protein L21 | 0.744222 | 0.02191 |
| K06950 | Uncharacterized protein | 0.754447 | 0.03272 |
| RpsL | 30S ribosomal protein S12 | 0.757252 | 0.0103 |
| RpmB | 50S ribosomal protein L28 | 0.757396 | 0.005197 |
| QueA | S-adenosylmethionine:tRNA ribosyltransferase-isomerase | 0.758379 | 0.01711 |
| RpsK | 30S ribosomal protein S11 | 0.758466 | 0.000854 |
| RpsJ | 30S ribosomal protein S10 | 0.76435 | 0.000912 |
| RplX | 50S ribosomal protein L24 | 0.764912 | 0.000767 |
| GntK | Gluconokinase | 0.767289 | 0.01813 |
| RpmD | 50S ribosomal protein L30 | 0.768817 | 0.009678 |
| E3.1.3.48 | Low molecular weight protein tyrosine phosphatase | 0.769773 | 0.03918 |
| ------ | Probable transcriptional regulatory protein B8W88_05570 | 0.774432 | 0.003453 |
| ------ | Uncharacterized protein conserved in bacteria | 0.774525 | 0.01996 |
| RplK | 50S ribosomal protein L11 | 0.775948 | 0.000886 |
| ------ | Beta-1,6-galactofuranosyltransferase | 0.781022 | 0.03658 |
| ------ | Uncharacterized protein | 0.782631 | 0.005339 |
| RplN | 50S ribosomal protein L14 | 0.786539 | 0.007537 |
| RplI | 50S ribosomal protein L9 | 0.787724 | 0.01191 |
| FtsY | Signal recognition particle protein | 0.789851 | 0.0274 |
| ------ | Uncharacterized protein | 0.790067 | 0.02148 |
| RpmJ | 50S ribosomal protein L36 | 0.790803 | 0.02758 |
| ------ | Uncharacterized protein | 0.792129 | 0.008607 |
| YtdB | Cell division initiation protein DivIVA | 0.79249 | 0.004057 |
| ------ | Uncharacterized protein | 0.794898 | 0.0132 |
| ------ | Transcriptional regulator | 0.799209 | 0.004459 |
| RpsS | 30S ribosomal protein S19 | 0.801295 | 0.000709 |
| RplQ | 50S ribosomal protein L17 | 0.801485 | 0.002255 |
| K09962 | Uncharacterized protein | 0.801742 | 0.03652 |
| YsiG | Putative endonuclease containing a URI domain | 0.805514 | 0.000703 |
| LocZ | Mid-cell-anchored protein Z | 0.806667 | 0.02129 |
| DhaL | Phosphoenolpyruvate-dihydroxyacetone phosphotransferase ADP-binding subunit DhaL | 0.80754 | 0.002646 |
| RplO | 50S ribosomal protein L15 | 0.809187 | 0.007542 |
| CshB | DEAD-box ATP-dependent RNA helicase CshB | 0.811146 | 0.0282 |
| ------ | Initiation-control protein YabA | 0.811881 | 0.007607 |
| ------ | Co-activator of prophage gene expression LbrB | 0.812376 | 0.01112 |
| YlxR | Uncharacterized protein | 0.815789 | 0.02921 |
| GalF | UTP--glucose-1-phosphate uridylyltransferase | 0.816244 | 0.01261 |
| ------ | Zn-dependent hydrolase (Beta-lactamase superfamily) | 0.816367 | 0.002553 |
| FabZ | 3-hydroxyacyl-[acyl-carrier-protein] dehydratase FabZ | 0.8166 | 0.002291 |
| ------ | GNAT family acetyltransferase | 0.816937 | 0.009269 |
| DapD | Maltose O-acetyltransferase | 0.819265 | 0.04962 |
| EngB | Probable GTP-binding protein EngB | 0.820398 | 0.01463 |
| HupB | Bacterial nucleoid DNA-binding protein | 0.820801 | 0.02339 |
| ------ | SWF/SNF family helicase | 0.821323 | 0.03454 |
| RpoZ | DNA-directed RNA polymerase subunit omega | 0.821705 | 0.0299 |
| NrdR | Transcriptional repressor NrdR | 0.822772 | 0.006565 |
| RplD | 50S ribosomal protein L4 | 0.824004 | 0.01276 |
| RluB | Pseudouridine synthase | 0.824159 | 0.01328 |
| RplS | 50S ribosomal protein L19 | 0.824597 | 0.006055 |
| YhbY | RNA binding protein | 0.824695 | 0.01838 |
| ------ | Resolvase | 0.824926 | 0.01877 |
| ParB | ParB protein | 0.82515 | 0.003081 |
| ------ | Accessory Sec system protein Asp2 | 0.826442 | 0.03672 |
| RpsC | 30S ribosomal protein S3 | 0.827569 | 0.003246 |
| GreA | Transcription elongation factor GreA | 0.827941 | 0.02114 |
| RpoE | Probable DNA-directed RNA polymerase subunit delta | 0.82811 | 0.01409 |
| ------ | Uncharacterized protein | 0.828263 | 0.01365 |
| Dut | Deoxyuridine 5'-triphosphate nucleotidohydrolase | 0.829171 | 0.000888 |
